# The Freshwater Cyanobacterium *Synechococcus elongatus* PCC 7942 Does Not Require an Active External Carbonic Anhydrase

**DOI:** 10.3390/plants13162323

**Published:** 2024-08-20

**Authors:** Elena V. Kupriyanova, Maria A. Sinetova, David A. Gabrielyan, Dmitry A. Los

**Affiliations:** K.A. Timiryazev Institute of Plant Physiology, Russian Academy of Sciences, 127276 Moscow, Russia; sinetova@ifr.moscow (M.A.S.); gabrielyanda@ifr.moscow (D.A.G.); losda@ippras.ru (D.A.L.)

**Keywords:** carbonic anhydrase, cyanobacteria, EcaA, physiological role, *Synechococcus elongatus* PCC 7942, *Cyanothece* sp. ATCC 51142, CO_2_-concentrating mechanism, extreme CO_2_ level, NDH-1_4_

## Abstract

Under standard laboratory conditions, *Synechococcus elongatus* PCC 7942 lacks EcaA^Syn^, a periplasmic carbonic anhydrase (CA). In this study, a *S. elongatus* transformant was created that expressed the homologous EcaA^Cya^ from *Cyanothece* sp. ATCC 51142. This additional external CA had no discernible effect on the adaptive responses and physiology of cells exposed to changes similar to those found in *S. elongatus* natural habitats, such as fluctuating CO_2_ and HCO_3_^−^ concentrations and ratios, oxidative or light stress, and high CO_2_. The transformant had a disadvantage over wild-type cells under certain conditions (Na^+^ depletion, a reduction in CO_2_). *S. elongatus* cells lacked their own EcaA^Syn^ in all experimental conditions. The results suggest the presence in *S. elongatus* of mechanisms that limit the appearance of EcaA^Syn^ in the periplasm. For the first time, we offer data on the expression pattern of CCM-associated genes during *S. elongatus* adaptation to CO_2_ replacement with HCO_3_^−^, as well as cell transfer to high CO_2_ levels (up to 100%). An increase in CO_2_ concentration coincides with the suppression of the NDH-1_4_ system, which was previously thought to function constitutively.

## 1. Introduction

Carbonic anhydrase (CA, EC 4.2.1.1) is the enzyme that maintains the equilibrium concentrations of two forms of inorganic carbon (C_i_) based on the pH of the environment: CO_2_ + H_2_O ⇆ H^+^ + HCO_3_^−^ (pKa ~ 6.36). Components of the CA reaction are present in cells of all organisms of carbon-based life. This explains the extraordinary occurrence of CA in nature. The enzyme is involved in a wide range of biological processes that require the acceleration of CO_2_/HCO_3_^−^ interconversions or a rapid change in the concentration of one of the four reaction components.

CAs have been divided into eight classes (α, β, γ, δ, ζ, η, θ and ι) based on their catalytic characteristics, amino acid sequence, spatial structure, and active site organization [1]. Cyanobacteria possesses three types of CAs: α, β, and γ. In model freshwater and marine species, these enzymes are found in carboxysomes, associated with thylakoid membranes, or in the cell’s outer layers external to the cytoplasmic membrane (CM) [2,3,4,5,6].

The physiological significance of cyanobacterial intracellular (carboxysomal and thylakoid) CAs is determined by their involvement in the operation of the CO_2_-concentrating mechanism (CCM), which enhances photosynthetic carbon fixation efficiency in the Calvin cycle [5,7,8]. The intracellular pool of HCO_3_^−^ in CCM is formed with the participation of (1) three bicarbonate transporters—BCT1 (bicarbonate transporter 1), SbtA (sodium-bicarbonate transporter A), and BicA (bicarbonate transporter A), and (2) two CO_2_ uptake systems (NDH-1_3/4_) that represent special modification of NADPH dehydrogenase (NDH-1) complexes [9]. Sun et al. [5] suggested that the thylakoid form of β-CA EcaB has a role in NDH-1_3/4_ function. HCO_3_^−^, which accumulates in the cytoplasm, is transformed into CO_2_ by carboxysomal CAs located near the active site of ribulose-1,5-bisphosphate carboxylase/oxygenase. In different species of cyanobacteria, carboxysomal CAs include CcaA (carboxysomal carbonic anhydrase A) and CsoSCA (carboxysome shell carbonic anhydrase) proteins of the β-class as well as CcmM (carbon concentrating mechanism protein M) of the γ-class [4].

Unlike their internal counterparts, external CAs have unknown physiological roles. In freshwater and marine model strains, these CAs are represented by EcaA and EcaB proteins (external carbonic anhydrase) of α- and β-class, respectively. Early studies of EcaA/B have shown that they do not participate in CCM operations [2,10]. The questionable involvement of EcaA/B in physiological processes was compounded by the fact that their catalytic activity had not been demonstrated at that time. It has been suggested that EcaA/B may deliver CO_2_/HCO_3_^−^ for the C_i_ transporters, act as sensors to detect CO_2_ levels in the environment or control CO_2_ leakage from cells [2,10]. However, these assumptions have not yet been confirmed. The recent identification of specific CA activity in EcaA/B [5,6,11] motivated us to explore the question of its physiological importance.

EcaA/B location within the cell’s outer layers is conditioned by the presence of a signal peptide region at the *N*-terminus of their amino acid sequence for transfer through the CM. In addition, EcaB has a putative lipoprotein lipid attachment site [10]. Despite the presence of the *ecaA* and *ecaB* genes in many cyanobacterial genomes, the literature offers just a few observations on the activity, localization, and potential biological roles of the corresponding proteins highlighted below.

Only two cyanobacteria have been shown to have EcaA in their outer layers: freshwater *Anabaena* sp. PCC 7120 [2] and marine *Cyanothece* sp. ATCC 51142 [6]. The latter is also known as *Crocosphaera subtropica* [12]. Early research found EcaA in total proteins of *Synechococcus elongatus* PCC 7942 [2], but this was later refuted [11].

EcaA in *Anabaena* was not further investigated. In *Cyanothece*, the EcaA protein (EcaA^Cya^) ensures relatively high external CA activity [6]. Its translocation through CM is provided by the Sec (secretory) export system [13]. The recombinant EcaA^Cya^ lacks redox regulation of activity, a characteristic feature of α-CAs. It should be emphases that EcaA^Cya^ is the only known α-class CA responsible for the external activity of cyanobacterial cells. However, the physiological significance of this protein in *Cyanothece* remains unclear.

The *Cyanothece* genome also contains a gene for a second external CA, EcaB. Unlike EcaB^6803^ of *Synechocystis* sp. PCC 6803 [5] (see below), the specific enzyme activity of EcaB^Cya^ has not been verified [6], raising the question of whether this protein serves a distinct biological function.

The enzymatic activity of the single external CA of freshwater *S*. *elongatus* PCC 7942, EcaA^Syn^, was demonstrated only in recombinant protein [11]. Unlike EcaA^Cya^, EcaA^Syn^ possesses an essential disulfide bond, which enables redox control of its activity. In retrospect, the failures of the early attempts to validate the enzymatic activity of this protein [2,10] could likely be attributed to the use of an ineffective expression system and/or isolation buffer containing reducing agents, such as dithiothreitol. It is worth mentioning that when EcaA^Syn^ is expressed heterologously in *E*. *coli*, the recombinant full-length protein remains inside the cells [11]. This could imply poor recognition of its signal peptide by the relevant bacterial Tat (twin-arginine translocation) export machinery.

*Synechococcus* cells possess a relatively low level of *ecaA^Syn^* transcript, which varies barely as the concentration of exogenous CO_2_ decreases from 1.5 to 0.04% [11]. As noted above, the presence of EcaA^Syn^ in *Synechococcus* is not evident, and intact cyanobacterial cells lack external CA activity. In this regard, it is not conceivable to discuss EcaA^Syn^’s physiological role under conventional laboratory culture conditions.

*Synechocystis* sp. PCC 6803 genome has only one external CA gene, *ecaB*. The presence of EcaB^6803^ in the periplasmic space of *Synechocystis* was initially directly confirmed using proteomics [3]. The presence of the twin-arginine motif in the signal peptide of EcaB^6803^ suggested that the Tat system was involved in its export through CM. However, further investigation revealed that the majority of EcaB^6803^ is associated with thylakoid membranes, with only a minor portion of the protein allocated to the CM [5]. As mentioned above, the biological function of EcaB^6803^ thylakoid form is linked to the operation of CO_2_-uptake systems NDH-1_3/4_ [5], while its role in the periplasm remains unknown. There have been no reports on the CA activity of native *Synechocystis* cells.

In addition to studies on model cyanobacterial strains, multiple investigations have found active external CAs in a wide range of alkaliphilic and haloalkaliphilic species [14,15,16]. However, in most cases, this activity has not been linked to any particular proteins. It is assumed that in dense cyanobacterial mats, where impeded diffusion of C_i_ may restrict photosynthetic efficiency, these CAs prevent CO_2_ leakage from cells.

Globally, freshwater, marine, and alkaliphilic cyanobacteria cells appear to have distinct requirements for the presence of active CAs in their outer layers. The pattern of participation of these enzymes in the photosynthetic assimilation of C_i_ may vary, being directly reliant on its exogenous level and the prevailing available form (CO_2_/HCO_3_^−^), determined by the pH of the environment.

The present study focuses on evaluating the role of external CA in the freshwater *S*. *elongatus* strain PCC 7942. We generated a series of cyanobacterial transformants that constitutively express distinct EcaA protein variations (Table 1). For the physiological tests, the transformant with full-length EcaA protein from *Cyanothece* sp. ATCC 51142, with its own signal peptide (L^Cya^-EcaA^Cya^), was selected. Here, we designated it as “TF”.

Physiological processes in the periplasm that might require external CA should be accompanied by variations in CO_2_, HCO_3_^−^, or H^+^ concentrations, all of which are components of the enzyme-catalyzed reaction. CA’s primary physiological role in cyanobacterial cells has traditionally been attributed to photosynthetic assimilation of C_i_ [8,18,19]. Despite previous evidence that external CAs play no role in CCM function [2,10], we cannot rule out the possibility that the enzyme plays a role in the so-called “basal” state [20] of this mechanism. From this perspective, the most evident role for CA located in the periplasm may be to supply CO_2_/HCO_3_^−^ molecules for their transport through the CM. In addition, the enzyme may be involved in maintaining the cell’s Na^+^/H^+^ balance, which is strongly related to C_i_ consumption [9].

To explore differences in the physiology of wild-type and TF cells, a series of experiments were performed to mimic fluctuations that occur in the natural environment of *Synechococcus*. The most crucial factors here are fluctuations in the hydrochemical properties of the environment and the resulting changes in the conditions of C_i_ supply. Such change may be caused by soil leaching and water enrichment with HCO_3_^−^ ions, resulting in an increase in the pH of the environment to alkaline levels. Fluctuations in hydrochemical parameters can also occur as a result of the reverse process, desalination, which reduces the concentration of Na^+^ ions required for the Na^+^-dependent consumption of HCO_3_^−^. During this series of experiments, our primary focus was on monitoring growth parameters as well as changes in the expression level of systems for photosynthetic C_i_ uptake and maintaining the cell’s Na^+^/H^+^ balance, which was associated with changes in the periplasm concentration of molecules included in the CA reaction equation—CO_2_, HCO_3_^−^, and H^+^.

In addition, we tested the validity of two additional hypotheses about the role of external CAs. The first concept centered on the enzymes’ putative protective role at extremely high exogenous concentrations of CO_2_ [21]. Previously, we discussed that external CAs may be an artifact of so-called pre-CCM, which operated in an early Earth’s CO_2_-rich atmosphere. The physiological role of CA at that time may have been to counteract the unlimited entry of CO_2_ into the cell by converting its major flux into HCO_3_^−^, followed by the uptake of bicarbonate ions by low-affinity transporters in amounts sufficient for photosynthesis.

Another question was whether EcaA, similarly to α-CA III in mammals [22], can be involved in the cellular response to oxidative stress. This function is due to the presence of two reactive cysteine sulfhydryl groups in α-CAs, which, in most cases, condition the ability of the enzyme to redox-regulate the activity. However, this characterization is also capable of conditioning the antioxidant properties of the molecule in analogy to glutathione, a key agent for resistance to oxidative stress in many living organisms, including plants. Like CA III, EcaA^Cya^ possesses two cysteine residues (Cys^55^ and Cys^209^ in the sequence of full-length protein). Despite the presence of thiol groups, redox status had no effect on enzyme functionality [6]. It appears that due to the presence of Cys^55^ and Cys^209^, EcaA^Cya^ can neutralize or mitigate the action of oxidizing agents. In photosynthetic organisms living in natural environments, oxidative stress can arise as a result of a sudden increase in light intensity, causing an imbalance in the reactions of the light and dark phases of photosynthesis and the subsequent accumulation of reactive oxygen species (ROS) in cells.

According to our findings, freshwater *Synechococcus* typically does not require the presence of an active external CA under all of the examined conditions. Our results also point to the mechanisms that prevent the periplasmic appearance of the active EcaA^Syn^ in *Synechococcus*. It seems that this scenario resulted from the evolutionary reduction in *Synechococcus* of the mechanisms that assure the appearance of EcaA^Syn^ in the cell.

## 2. Results and Discussion

### 2.1. Generation of Synechococcus Transformants with Constitutive Expression of External CAs and Assessing the Presence of Specific mRNA

Several transformants of *S*. *elongatus* PCC 7942 with constitutive expression of external CA proteins have been generated (Table 1). The target CAs were expressed in *Synechococcus* cells using the *trc* promoter, which ensures constitutive protein production in cyanobacterial cells regardless of growing circumstances. The pAM1303 vector used for transformation resulted in double homologous recombination of the cloned DNA fragment into a neutral region of the *Synechococcus* genome [23]. Figure S1 shows PCR results demonstrating the insertion of target nucleotide sequences into the *Synechococcus* genome and the segregation of modified chromosomes.

It should be noted that replacing the native copy of the EcaA^Syn^ gene within the *Synechococcus* chromosome via homologous recombination was irrational for two reasons: (1) The Synpcc7942_1389 gene, which encodes the D1 protein of photosystem II, is located right next to *ecaA^Syn^* (Synpcc7942_1388); (2) the native copy of *ecaA^Syn^* is unable to assure the synthesis of EcaA^Syn^ protein in *Synechococcus* cells [11].

**Figure 1:**
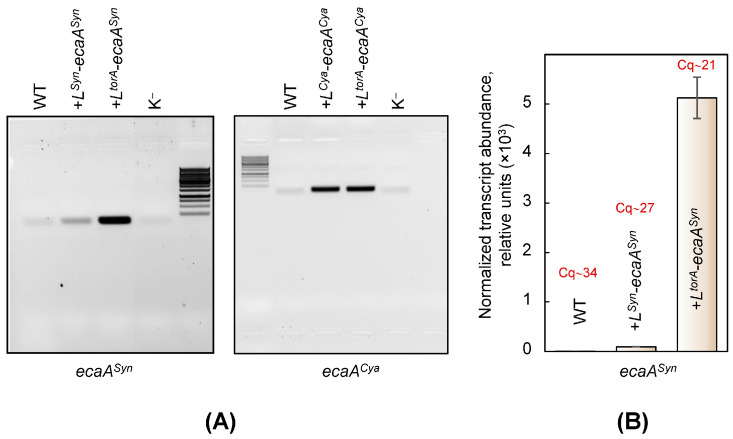
Detection of mRNA corresponding to CA target sequences in *Synechococcus* transformants across different lines. PCR results on a cDNA template prepared by reverse transcription following total RNA isolation. (**A**) Semi-quantitative RT-PCR with gene-specific primers for the *ecaA^Syn^* and *ecaA^Cya^* genes. Electrophoresis on a 2% agarose gel shows the amount of product accumulated throughout 25 reaction cycles. DNA was visualized with ethidium bromide (inverted picture). (**B**) RT-qPCR results utilizing primers for the *ecaA^Syn^* for transformants carrying the constructs *L^Syn^-ecaA^Syn^* and *L^torA^-ecaA^Syn^*. The graphs represent the level of expression of the *ecaA^Syn^* gene in transformants relative to that of the wild type (WT); the last was referred to as 1. Data are normalized to the expression level of the *secA* gene. Abbreviations: K^−^—negative control, a PCR mixture with no DNA template.

Figure 1A depicts the results of semi-quantitative PCR, which demonstrate the presence of mRNA encoding recombinant proteins in all transformants. The results of real-time PCR confirm these data (Figure 1B). Wild-type Cq values (~34) reflect the limit of reliable mRNA content. Although the amount of *L^Syn^*-*ecaA^Syn^* gene transcripts in the transformant was higher than in wild-type cells (100:1), it was still much lower than what can be achieved through the expression controlled by the *trc* promoter. Particularly, it was substantially lower compared to the transformant carrying the *L^torA^*-*ecaA^Syn^* sequence. One possible explanation for the small amount of *L^Syn^*-*ecaA^Syn^* mRNA is the existence of a specific tag in its sequence for intracellular nuclease(s).

### 2.2. Confirmation of Recombinant Proteins’ Presence in Synechococcus Transformants and Their External CA Activity

**Figure 2:**
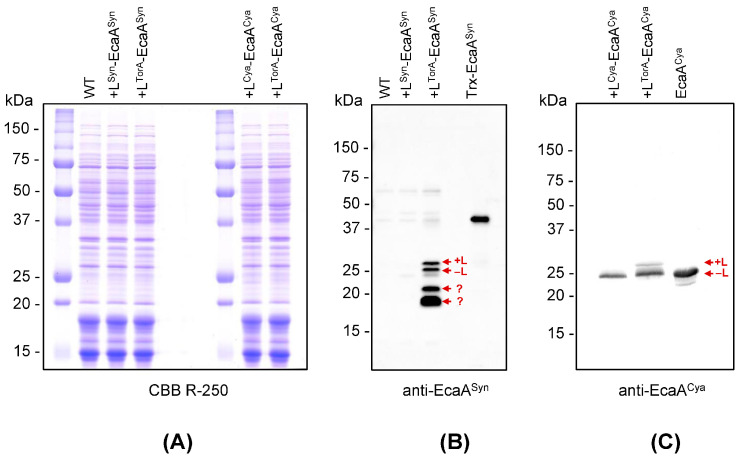
Assess the presence of target recombinant proteins in *Synechococcus* transformants expressing the L^Syn^-EcaA^Syn^, L^TorA^-EcaA^Syn^, L^Cya^-EcaA^Cya^, and L^TorA^-EcaA^Cya^ proteins. (**A**) Electrophoretic separation of soluble proteins (including proteins from the periplasmic space) in denaturing 12.5% PAGE stained with Coomassie brilliant blue R-250. Each lane included 7.5 μg of protein. (**B**,**C**) Western blot analysis of soluble proteins using antibodies to recombinant EcaA^Syn^ or EcaA^Cya^. Positive controls include recombinant proteins Trx-EcaA^Syn^ (EcaA^Syn^ fused to thioredoxin at its *N*-terminus) and EcaA^Cya^, which were applied at a concentration of 2 ng per lane. The Figures show the positions of full-length proteins with a signal peptide (+L) (most likely located in the cytoplasm) and mature forms that are generated after transfer through the CM into the periplasmic space and removing the signal peptide (-L). Calculated molecular weight of proteins: EcaA^Syn^—24.2 kDa, L^Syn^-EcaA^Syn^—27.0 kDa, L^TorA^-EcaA^Syn^—28.7 kDa, EcaA^Cya^—26.5 kDa, L^Cya^-EcaA^Cya^—29.4 kDa, L^TorA^-EcaA^Cya^—31 kDa.

The presence of recombinant proteins in the soluble protein fraction of *Synechococcus* (enriched in cytoplasmic and periplasmic proteins) was clearly detected in transformants expressing L^TorA^-EcaA^Syn^, L^Cya^-EcaA^Cya^, and L^TorA^-EcaA^Cya^ (Figure 2).

The entire soluble L^Cya^-EcaA^Cya^ protein exists in its mature processed form (EcaA^Cya^). Because signal peptidases exclusively operate in the periplasmic space [24], this finding suggests that recombinant L^Cya^-EcaA^Cya^ translocated remarkably well through the *Synechococcus* CM. The substitution of the signal peptide in EcaA^Cya^ from the original one (L^Cya^) to L^TorA^ resulted in a portion of the recombinant L^TorA^-EcaA^Cya^ remaining as a full-length, non-processed protein. This finding suggests that L^TorA^-EcaA^Cya^ has lower translocation efficiency into the periplasmic space compared to L^Cya^-EcaA^Cya^.

L^TorA^-EcaA^Syn^ was also found in two forms in the transformant cells: processed and non-processed. Western blot analysis identified additional specific signals from polypeptides with molecular weights of less than 25 kDa that likely do not correspond to post-translationally modified EcaA^Syn^. These signals are most likely generated by intracellular peptidases degrading EcaA^Syn^ in the transformant’s cytoplasm.

The western blot pattern for the transformant, which expressed L^Syn^-EcaA^Syn^, was similar to that of the wild-type cells, with no distinguished signals detected. This is completely compatible with the findings, which indicate the extremely low level of the relevant mRNA in the cells of this transformant (Figure 1).

**Figure 3:**
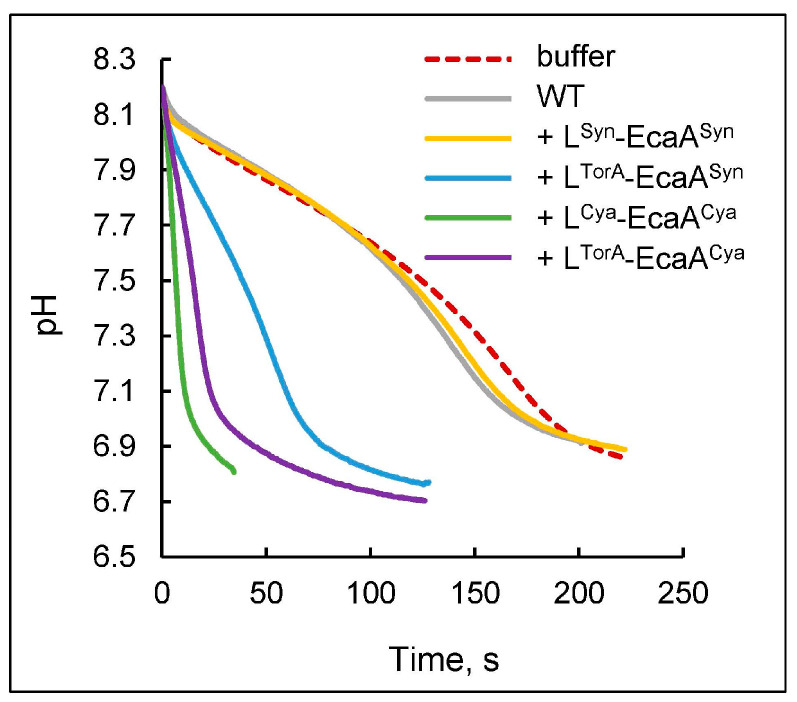
Assessment of CA activity in intact wild-type *Synechococcus* cells and transformants expressing L^Syn^-EcaA^Syn^, L^TorA^-EcaA^Syn^, L^Cya^-EcaA^Cya^ and L^TorA^-EcaA^Cya^ proteins. Measurements were carried out in 3–5 duplicates. The graphs depict the average curve for each sample.

External CA activity in transformants was evaluated, confirming the presence of an active enzyme in cells expressing the proteins L^TorA^-EcaA^Syn^, L^Cya^-EcaA^Cya^, and L^TorA^-EcaA^Cya^ (Figure 3). This activity clearly matches the processed forms of proteins in the periplasm, as evidenced in the western blot pattern (Figure 2). Visible differences in the slopes of equilibrium curves for wild-type cells and transformant with L^Syn^-EcaA^Syn^ do not appear to result from the enzymatic reaction: the rate of equilibrium, in this case, did not correlate with the number of cells introduced into the reaction and did not change with the addition of a specific CA inhibitor, ethoxyzolamide.

Summarizing the data presented in Figure 1, Figure 2 and Figure 3, we can conclude the following. External CAs were successfully expressed in *Synechococcus* cells only when they were different from their own EcaA^Syn^. We assume that *Synechococcus* possesses the intracellular mechanism that is specially targeted at preventing the appearance of EcaA^Syn^ at both the transcript and protein product levels.

Quantitative analysis revealed that intact cells of the *Synechococcus* transformant with constitutive expression of the L^Cya^-EcaA^Cya^ protein exhibit the highest external CA activity (Table 2). In this transformant, all recombinant CA was mature and processed (Figure 2C), indicating a very high efficiency of translocation through the CM. This circumstance is critical for reducing the risks of the so-called “short circuit” caused by the presence of CA in the cytosol [25], as such cells might be unable to perform efficient photosynthesis due to the efflux of accumulated C_i_ back into the environment. This is particularly important in the case of L^TorA^-EcaA^Cya^, which can function even in the reduced environment of cytoplasm due to the absence of redox control of its activity [6]. Thus, for subsequent physiological studies, we selected a transformant that expressed the L^Cya^-EcaA^Cya^ protein. In the following article, we shell refer to this transformant as TF.

### 2.3. Contribution of the External CA to Physiological Responses of Synechococcus When CO_2_ Is the Main Source of C_i_

Cyanobacteria can utilize both CO_2_ and the bicarbonate ion, HCO_3_^−^, as an exogenous C_i_ for photosynthesis [20]. The Henderson–Hasselbach equation, pH = 6.3 + lg([HCO_3_^−^]/[CO_2_]), directly determines the equilibrium ratio of the concentrations of these two types of C_i_ in the environment. At the same time, the cells always have access to CO_2_, which is present in aquatic environments at an equilibrium concentration with air [26].

The CO_2_ molecule and the HCO_3_^−^ ion differ significantly in their physicochemical properties; therefore, cyanobacterial cells use different strategies for their consumption [8,20]. CO_2_ can enter the cell by direct diffusion due to its high solubility in lipids. Cyanobacteria use the so-called “facilitated CO_2_ uptake” strategy, in which the entrance of these molecules is facilitated by the establishment of their negative gradient due to the quick conversion of CO_2_ that has already entered the cell into HCO_3_^−^. Unlike lipophilic CO_2_, negatively charged HCO_3_^−^ can cross cell membranes only via active transport. Energy equivalents for this process can be either ATP molecules or an electrochemical gradient of Na^+^ ions. In this aspect, CO_2_ consumption is preferable because the cell does not need to expend additional energy resources to obtain it.

If the periplasmic CA may supply CO_2_ and HCO_3_^−^ molecules for transport across the CM, the L^Cya^-EcaA^Cya^ should contribute to the C_i_ uptake into TF cells. This fact should be reflected in the expression patterns of the associated assimilation systems. At the same time, the TF’s advantage over the wild type in certain conditions should correlate with the physiological parameters (higher growth rate, biomass accumulation, etc.).

#### 2.3.1. Cultures Growth and Transcriptional Regulation of C_i_ Uptake Systems in Response to Changes in CO_2_-Supply

**Figure 4:**
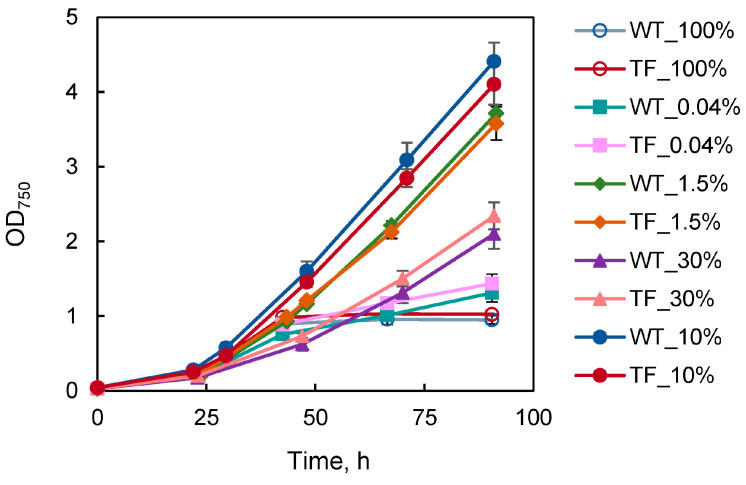
Growth curves for *Synechococcus* wild-type (WT) and transformant with constitutive expression of the L^Cya^-EcaA^Cya^ protein (TF) at different CO_2_ concentrations in the gas–air mixture. The switch from the standard CO_2_ concentration (1.5%) to medium (10%) and high (30%) values occurred 24 h after the start of cultivation, while the switch to low (0.04%) and extremely high (100%) values occurred on the second day. The graphs represent standard deviations from the mean of three biological replicates.

The phenotypes of wild-type and TF cells were compared using their growth curves at varied CO_2_ concentrations in the gas–air mixture (Figure 4). No statistically significant differences have been found between these two types of cells. Notably, at 10% CO_2_, the cultures showed slightly better growth rates than under standard conditions (1.5%). It should be noted that bubbling with 30 and 100% CO_2_ resulted in a pH drop in the culture medium from 7.5 to 6.5 and 6.0, respectively, by the end of the first hour after the commencement of adaptation, despite the presence of a buffer agent (HEPES-NaOH, pH 7.5). Thus, the cells suffered an additional nonspecific stress caused by acidification [27,28]. Particularly, under 100% CO_2_, culture growth significantly declined (Figure 4), and the pigments absorption spectra changed, reflecting a drop in the amount of chlorophyll and carotenoids.

The only components of the cyanobacterial CCM that are transcriptionally regulated in response to variations in the level of exogenous C_i_ are C_i_ assimilation systems [29,30,31,32]. *S*. *elongatus* PCC 7942 contains two high-affinity HCO_3_^−^ transporters, BCT1 and SbtA, as well as two CO_2_ uptake systems, low-affinity NDH-1_4_ and high-affinity NDH-1_3_. High-affinity systems are only expressed at low exogenous C_i_ concentrations (≤100 µM), which are insufficient for effective photosynthesis [33]. During conventional laboratory cultivation, C_i_-limiting conditions correspond to growth at or below the ambient CO_2_ concentration (0.04%).

**Figure 5:**
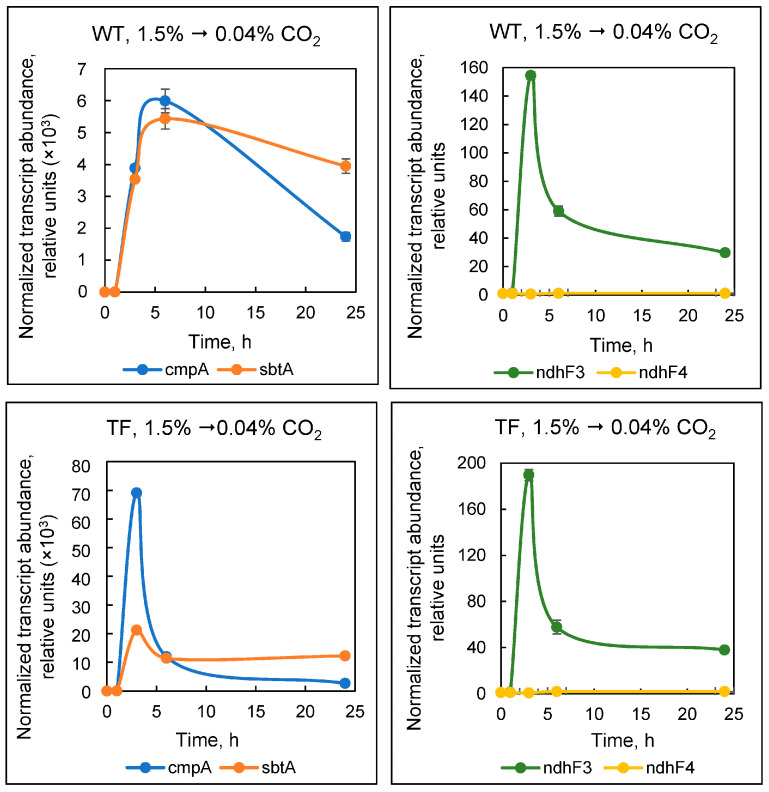
The dynamics of changes in the mRNA levels of genes related to C_i_ uptake systems in *Synechococcus* wild-type (WT) cells and that of transformant with constitutive expression of the L^Cya^-EcaA^Cya^ protein (TF) when the CO_2_ content in the gas–air mixture was immediately changed from 1.5 to 0.04% (without medium replacement). The expression level is presented in comparison to that at the zero-hour point, which corresponds to cell growth at 1.5% CO_2_ just before being transferred to low carbon dioxide concentration. The data are normalized to the expression level of the *petB* gene.

After switching the wild-type cell culture from 1.5% to 0.04% CO_2_, the maximal level of mRNA for the *sbtA* and *cmpA* genes (the latter encodes one of the BCT1 complex subunits) was attained by the sixth hour of adaptation (Figure 5): their transcript levels increased by approximately 6000 and 5400 times, respectively. By the third hour of adaptation, wild-type cells had attained the maximal amount of mRNA for the *ndhF3* gene, which encodes one of NDH-1_3_ subunits: it increased by 150 times relative to control conditions (1.5% CO_2_). The expression of *ndhF4*, which encodes one of the NDH-1_4_ proteins, changed by less than two-fold. In general, these results are consistent with previously known data [29].

When TF cells were switched from 1.5% to 0.04% CO_2_, a similar pattern emerged as in wild-type cells, with induction of the *cmpA*, *sbtA*, and *ndhF3* genes, but no changes in *ndhF4* expression. The difference was that the highest levels of *cmpA*, *sbtA*, and *ndhF3* transcripts were achieved during the third hour of adaptation (Figure 5). The mRNA levels of these genes grew by around 70,000, 20,000, and 190 times, respectively.

This might be related to the function of periplasmic EcaA^Cya^ as follows. At 1.5% CO_2_, carbon dioxide saturates the culture medium, and it is partially converted into HCO_3_^−^—the main form of C_i_ at pH 7.5 [34]. When cultures are switched to 0.04% CO_2_, EcaA^Cya^, which has access to the external substrate, rapidly transfers the reserves of dissolved HCO_3_^−^ into CO_2_, followed by the release of the latter out of the culture medium. This is also facilitated by intensive bubbling of the cell suspension. Therefore, TF cells sense a decrease in the amount of HCO_3_^−^ in the medium more quickly than the wild-type. This explains the earlier induction of HCO_3_^−^ uptake systems (BCT1 and SbtA) in the TF compared to the wild type, as well as the timing of induction of the CO_2_-uptake system NDH-1_3_. Thus, when exogenous CO_2_ concentrations suddenly drop, external CA activity becomes a disadvantage rather than a physiological priority.

**Figure 6:**
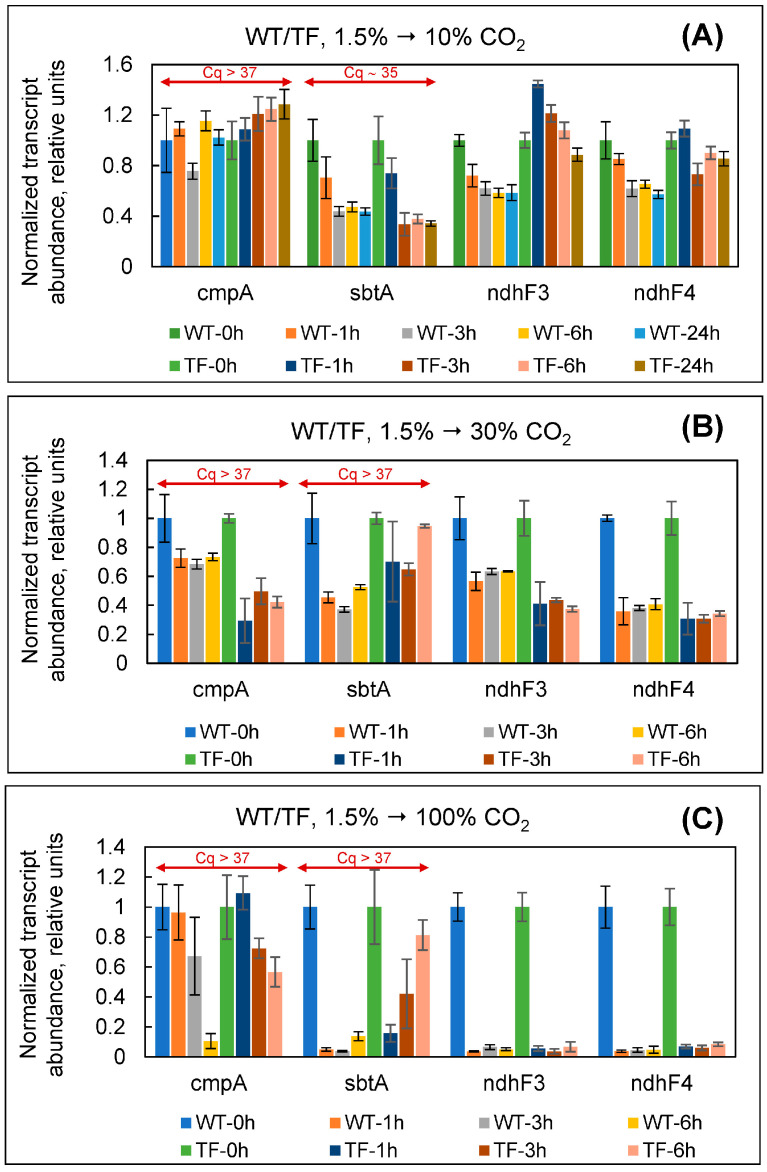
Dynamics of changes in the level of mRNA of genes associated with C_i_ uptake systems in *Synechococcus* wild-type (WT) cells and that of transformant with constitutive expression of the L^Cya^-EcaA^Cya^ protein (TF) when the CO_2_ content in the gas–air mixture changes from 1.5 to 10% (**A**), 30% (**B**), or 100% (**C**). The expression level is presented in comparison to that at the zero-hour point, which corresponds to cell growth at 1.5% CO_2_, just before transferring to different experimental conditions. The data is normalized to the expression level of *secA* (1.5 → 10%), *ivlD* and *secA* (1.5 → 30%), or *ilvD* (1.5 → 100%) genes.

When cultures were switched from 1.5% to 10% CO_2_, the amount of *ndhF3* and *ndhF4* genes mRNAs changed by less than twofold in both cell types, showing that their transcription was neither induced nor repressed (Figure 6A). The observed Cq values (~35) for *sbtA* indicated the limit of the reliable mRNA content. Most likely, under control (1.5% CO_2_) and experimental (10% CO_2_) conditions, the mRNA of this gene was absent. Similarly, for *cmpA*, the Cq value was greater than 37 at both 1.5% and 10% CO_2_, showing the absence of the specific transcript under both conditions.

In tests involving cultures transfer to 30 and 100% CO_2_, we did not explore the long-term adaptation responses since cells encountered clear non-specific stress due to the decrease in pH of the culture medium (see above). Under both experimental conditions (30 and 100% CO_2_), the Cq values for *cmpA* and *sbtA* were more than 37, indicating the lack of specific mRNA. When cultures were switched from 1.5% to 30% CO_2_, the expression levels of the *ndhF3* and *ndhF4* genes fell within one hour of the commencement of adaptation (Figure 6B). These transcript levels decreased 2.4 and 3.2 times in the wild-type and 1.8 and 2.8 times in the TF cells, respectively. The Cq values of 29–32 for *ndhF3* and *ndhF4* at 30% CO_2_ confirm the reliability of the data.

When cells were exposed to 100% CO_2_, the amounts of *ndhF3* and *ndhF4* mRNAs decreased even more significantly (Figure 6C): one hour after starting adaptation, wild-type cells experienced a drop of 27 and 26 times, respectively. In transformed cells, *ndhF3* and *ndhF4* transcript levels decreased by approximately 18 and 14 times, respectively. Both genes’ Cq values increased from ~31 (at 1.5% CO_2_) to >36 (at 100% CO_2_), indicating the complete elimination of the specific mRNAs at extremely high CO_2_ concentrations.

With the exception of slight variations in changes in *ndhF3* and *ndhF4* transcription levels at 30 and 100% CO_2_, the results shown in Figure 6 were comparable for wild-type and TF cells. In general, the data presented in Figure 5 and Figure 6 indicate that *Synechococcus* high-affinity bicarbonate uptake systems, BCT1 and SbtA, are activated exclusively at atmospheric CO_2_ concentrations. At C_i_ concentrations sufficient to saturate photosynthesis (in our case, 1.5% CO_2_ or more), the cyanobacterium uses CO_2_ in an energy-saving manner. At the same time, CO_2_-uptake systems NDH-1_3/4_ in *Synechococcus* are gradually inhibited as CO_2_ concentrations rise from natural to extremely high levels. The most striking discovery is the inhibition of the NDH-1_4_, which was previously assumed to be constitutive and whose expression is independent of the level of exogenous CO_2_ supply.

Simultaneously, we may infer that the gathered data do not support our hypothesis regarding the protective effect of external CAs in the conditions of an ancient CO_2_-rich atmosphere [21]. If the external CA helped to create a barrier that prevented unrestricted CO_2_ entry into the cell, the TF would have advantages over the wild type since it would experience less CO_2_ stress. In this instance, we would have detected a distinct change in the parameters presented in Figure 4 and Figure 6.

**Figure 7:**
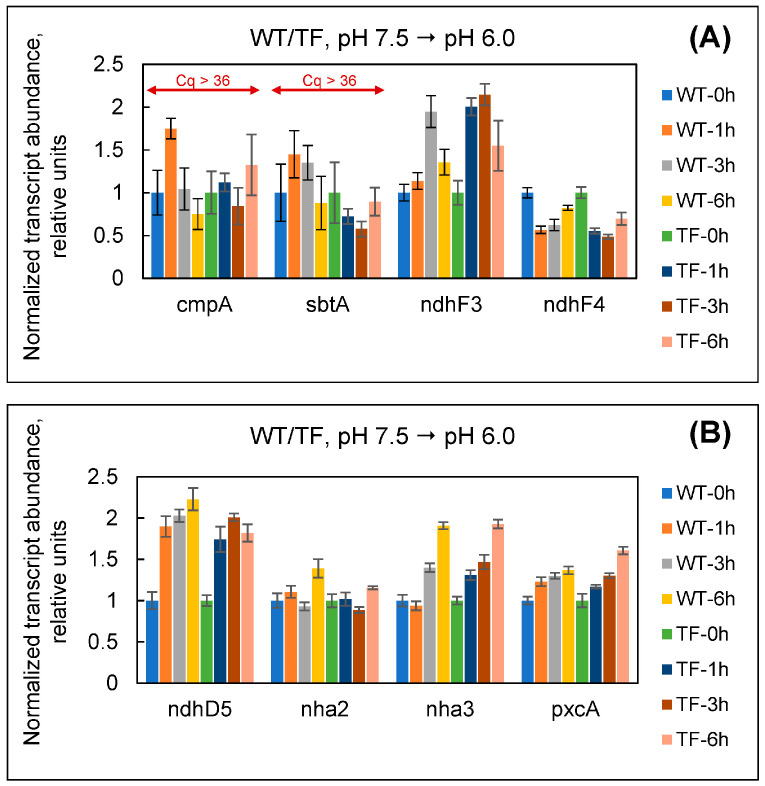
The dynamics of changes in the level of mRNA of genes associated with C_i_ uptake systems (**A**) and systems of Na^+^/H^+^ balance maintenance (**B**) in *Synechococcus* wild-type (WT) cells and that of transformant with constitutive expression of the L^Cya^-EcaA^Cya^ protein (TF) when transferred from a standard BG-11 medium (20 mM HEPES, pH 7.5) to BG-11 with 20 mM MES, pH 6.0. The level of gene expression is presented in comparison to that at the zero-hour point, which corresponds to cell growth on BG-11 with pH 7.5, just before being transferred to BG-11 with pH 6.0. Data are normalized on *secA* gene expression levels.

Since bubbling with high (30%) and extremely high (100%) CO_2_ concentrations caused a decrease in the pH of the culture medium to 6.5–6.0, we conducted an additional experiment to determine whether the changes observed in Figure 6B,C were specific. Wild-type and TF *Synechococcus* cells cultivated under conventional conditions (1.5% CO_2_, BG-11, 20 mM HEPES, pH 7.5) were transferred to BG-11 with pH 6.0 and 20 mM MES as a buffer, keeping the percentage of CO_2_ in the gas–air mixture unchanged. This allowed us to re-create acidification of the environment in response to excessive CO_2_ percentage while subtracting the CO_2_ stress factor itself. In this experiment, cells showed no significant changes in *cmpA*, *sbtA*, *ndhF3*, or *ndhF4* expression (Figure 7A). At the same time, *cmpA* and *sbtA* had Cq values greater than 36; Cq for *ndhF3* was ~34. This scenario was markedly different from that in Figure 6B,C, and, in terms of C_i_ assimilation system behavior, mirroring the state of cells when they were normally cultivated at 1.5% CO_2_ (repression of BCT1, SbtA, and NDH-1_3_; C_i_ assimilation through CO_2_-uptake system NDH-1_4_). Thus, it can be stated that transcriptional alterations in *ndhF3* and *ndhF4* in Figure 6B,C resulted from specific cell responses during adaptation to high and extremely high CO_2_ levels.

#### 2.3.2. Operation of Na^+^/H^+^-Balance Systems during C_i_ Assimilation under Different CO_2_-Supply Conditions

C_i_ assimilation by a cyanobacterial cell is directly linked to the maintenance of its Na^+^/H^+^ balance, in which periplasmic CA may play a role due to its ability to quickly adjust H^+^ concentration. The CM contains the following auxiliary elements that ensure the operation of C_i_ uptake systems in model strains of cyanobacteria: (a) Na^+^/H^+^ antiporter Nha, which contributes to the formation of a sodium ion gradient during Na^+^-dependent bicarbonate transport; (b) proton pump PxcA, which works to release H^+^ from the cytoplasm and maintains a constant pH in the cell in the slightly alkaline range; and (c) the specialized NDH-1 complex Mnh, which functions as a Na^+^/H^+^ antiporter or H^+^ pump [9].

A literature search [35] and a survey of the genome of *S*. *elongatus* PCC 7942 in Cyanobase (http://genome.microbedb.jp/cyanobase/, accessed on 19 July 2024) reveals the presence of the following components of the Na^+^/H^+^ balance system in this cyanobacterium: the Mnh complex (individual subunits encoded by the genes Synpcc7942_1468, Synpcc7942_1469, Synpcc7942_1473, and Synpcc7942_1474); potential Na^+^/H^+^ antiporters Nha1–7 (Synpcc7942_0811, Synpcc7942_1264, Synpcc7942_2359, Synpcc7942_0546, Synpcc7942_0307, Synpcc7942_2394, and Synpcc7942_2186); as well as the PxcA proton pump (Synpcc7942_0991).

The following genes were selected for this study: (1) *ndhD5* (Synpcc7942_1473), which encodes one of the Mnh complex’s subunits; (2) *nha2* (Synpcc7942_1264), and *nha3* (Synpcc7942_2359) for potential Na^+^/H^+^ antiporters, which have been chosen based on the data of Billini et al. [35]; and (3) pxcA (Synpcc7942_0991), which encodes the corresponding proton pump.

**Figure 8:**
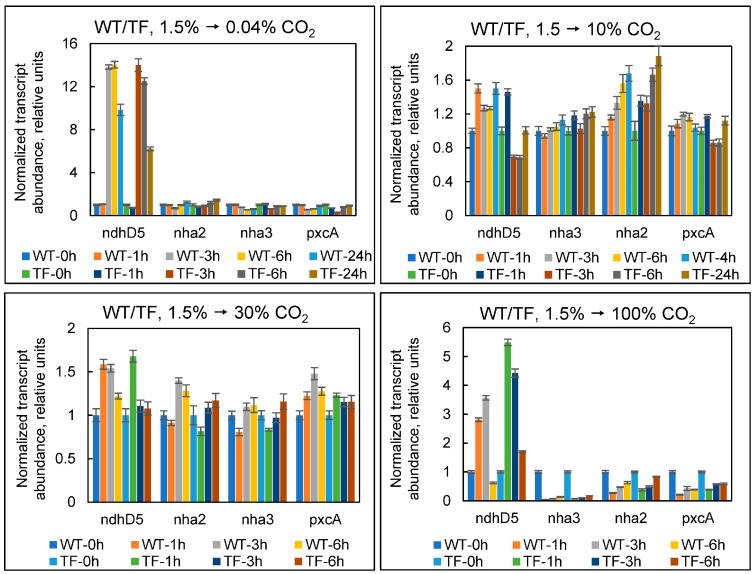
Changes in the mRNA levels of genes associated with Na^+^/H^+^ balance systems in *Synechococcus* wild-type (WT) cells and that of transformant with constitutive expression of the L^Cya^-EcaA^Cya^ protein (TF) when transferred from optimal growth conditions (1.5% CO_2_) to increased (10, 30, 100%) or reduced (0.04%) CO_2_ concentrations. The level of gene expression is presented in comparison to that at the zero-hour point, which corresponds to cell growth at 1.5% CO_2_, just before switching to different CO_2_ concentrations. The data is normalized to the expression level of the following genes: *petB* (1.5 → 0.04%), *petB* and *secA* or *petB* and *ivlD* (1.5 → 10%), *secA* and *ivlD* (1.5 → 30%), and *petB* and *ivlD* (1.5 → 100%).

Figure 8 shows the transcriptional response of the above-mentioned genes to variations in the concentration of exogenous CO_2_. The effects were identical in both wild-type and TF cells. When cultures were switched from 1.5 to 10 and 30% CO_2_, we observed no significant changes in the expression of any of the genes tested. The Mnh complex showed a clear rise in the expression level, both during a drop (from 1.5 to 0.04%) and an excessive increase (from 1.5 to 100%) in exogenous CO_2_. The induction of Mnh under 0.04% CO_2_ coincides with an increase in the expression of bicarbonate uptake systems under these conditions (Figure 5). The function of Mnh here is clearly related to the maintenance of Na^+^-dependent HCO_3_^−^ uptake by the SbtA transporter. Predicting Mnh’s physiological role at extremely high CO_2_ levels (100%) is challenging. It cannot be employed to counteract acidification because Mnh can only offer an intracellular H^+^ supply in exchange for Na^+^ ions. By the way, when the culture medium was intentionally acidified, we did not find a comparable increase in *ndhD5* transcript levels (Figure 7B).

When both cell types were exposed to extremely high CO_2_ levels (100%), the level of transcripts for all other examined genes (*nha2*, *nha3*, and *pxcA*) sharply decreased within one hour, paralleling the increase in *ndhD5* expression (Figure 8). Because there was no similar response to acidification of the environment (Figure 7B), we interpret Nha2/3 and PxcA suppression as a CO_2_ stress-specific response, the physiological impact of which is unknown. One can suppose that the repression of Nha2/3 and PxcA is due to the strong suppression of Na^+^-dependent bicarbonate transport at 100% CO_2_ (Figure 6, *sbtA* data). However, at 10 and 30% CO_2_, the levels of *nha2*, *nha3*, and *pxcA* transcripts remained unchanged, although the expression of SbtA was also suppressed.

We observed equivalent fluctuations in the expression levels of *ndhD5*, *nha2*, *nha3*, and *pxcA* in both cell types. Thus, the presence of external CA in the TF had no effect on the cell’s Na^+^/H^+^ balance during photosynthetic assimilation of C_i_ at the examined conditions.

### 2.4. Contribution of the External CA to Physiological Responses of Synechococcus When HCO_3_^−^ Is the Main Source of C_i_

*Synechococcus*, as a freshwater microorganism, can withstand relatively high concentrations of bicarbonate and the resulting alkaline pH value in its habitat. This conclusion can be derived from the findings of early investigations on *Synechococcus* species that are close to *S*. *elongatus* PCC 7942 [36,37,38].

In this series of experiments, the media where HCO_3_^−^ was the main carbon source was used (in contrast to cultivation on BG-11 under bubbling with CO_2_-containing gas–air mixture). As in previous cases, our goal was to look for differences between the physiological responses of TF and those of wild-type cells.

#### 2.4.1. Evaluation of *Synechococcus* Tolerance to Different HCO_3_^−^ Contents in the Culture Medium

To determine the appropriate HCO_3_^−^ amount in the culture medium for *Synechococcus* growth, we conducted three independent experiments in which wild-type and TF cells were grown in BG-11 with various concentrations of NaHCO_3_ (from 10 to 200 mM). NaHCO_3_ basic characteristics resulted in an initial pH~9.5 for all medium variants.

The spectral characteristics of the experimental cultures appeared normal and similar to those of the control cells (Figure S2). The maximum pigment content was found in cultures grown at 10–100 mM NaHCO_3_, which was consistent with their overall view (Figure S3). The alkalization of the environment in all variations with NaHCO_3_ obtained comparable values, implying that photosynthetic intensity was almost the same. The optimal NaHCO_3_ level for both cell types ranged between 10 and 50 mM. Under these conditions, the culture suspension density and biomass accumulation were at their peak (Figure S2). Meanwhile, we found no variations in the physiological responses to culture conditions in wild-type and TF cells.

#### 2.4.2. Transcriptional Response of *Synechococcus* Cells during Adaptation to Bicarbonate-Containing Media

**Figure 9:**
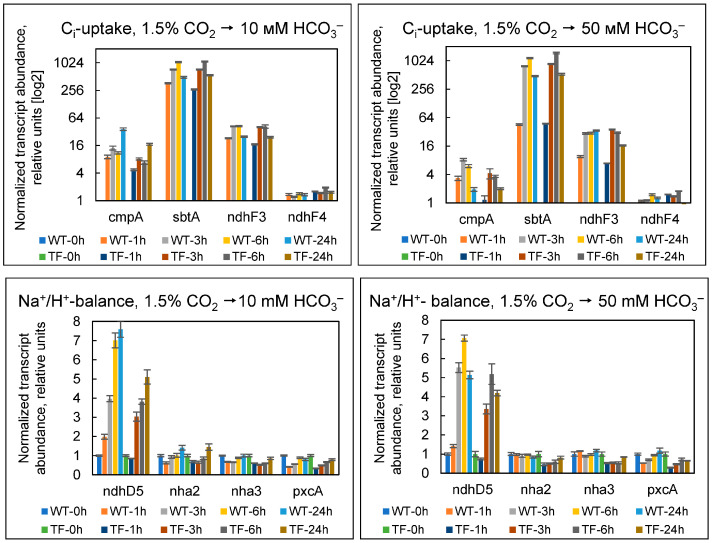
Changes in the level of transcripts of genes associated with the C_i_ uptake and Na^+^/H^+^ balance systems in *Synechococcus* wild-type (WT) cells and that of transformant with constitutive expression of the L^Cya^-EcaA^Cya^ protein (TF) when transferred from standard cultivation conditions (BG-11, pH 7.5, 1.5% CO_2_) to BG-11 with NaHCO_3_ (10 or 50 mM, pH 9.5). The level of gene expression is presented in comparison to that at the zero-hour point, which corresponds to cell growth under standard conditions, just before being transferred to NaHCO_3_-containing media. Data are normalized to the expression levels of the *ivlD* and *secA* genes.

In the experiments, we employed BG-11 media with 10 or 50 mM NaHCO_3_ as boundary values of this parameter to ensure optimal cyanobacterial growth (Figures S2 and S3). Daily assessment of the transcriptional response of genes associated with C_i_ uptake and Na^+^/H^+^ balance-maintaining systems under adaptation to bicarbonate-containing environments found no significant differences between the two cell types under both experimental settings (Figure 9).

Remarkably, the transcriptional response of cells to a switch from 1.5% CO_2_ to 10 or 50 mM bicarbonate (Figure 9) was identical to that observed in response to a drop in CO_2_ from the optimal (1.5%) to the atmospheric (0.04%) level (Figure 5). In both scenarios, transcripts of genes related to inducible HCO_3_^−^ and CO_2_ uptake systems (*cmpA*, *sbtA*, and *ndhF3*) were significantly up-regulated, as well as of *ndhD5*, which encodes one of the Mnh complex’s subunits and serves as an auxiliary element for SbtA’s operation. At the same time, the NDH-1_4_ CO_2_ uptake system, which exhibits constitutive behavior at low C_i_ levels, did not respond to the changes. Thus, the transfer of *Synechococcus* cells from 1.5% CO_2_ to bicarbonate media caused the detectable and convincing induction of the CCM.

It is thought that cyanobacteria assess the overall concentration of exogenous C_i_ (CO_2_ + HCO_3_^−^) and initiate CCM only when its level is insufficient to saturate the dark phase of photosynthesis [8,39]. For cyanobacteria, C_i_-limiting conditions are defined as a total C_i_ level of no more than 0.1 mM in the medium [33]. While bubbling, 1.5% CO_2_ corresponds to at least 2 mM of total dissolved C_i_ [40]. Thus, a transfer of cells from 1.5% CO_2_ to 10 and 50 mM NaHCO_3_ provides even more total C_i_, the conditions that do not imply CCM induction. Our findings suggest that a shift from CO_2_ to HCO_3_^−^ (without a simultaneous decrease in the total amount of C_i_) forces *Synechococcus* to re-arrange CCM architecture in order to restructure C_i_ consumption from CO_2_ to HCO_3_^−^. The induction of *cmpA* and *sbtA* expression (Figure 9) appears to be linked to the necessity for the synthesis of the HCO_3_^−^ transporters BCT1 and SbtA. The activation of the CO_2_-uptake system NDH-1_3_ indicates that cells detect a decrease in the exogenous amount of a specific form of C_i_ (in this case, CO_2_) but not in the overall sum of CO_2_ + HCO_3_^−^.

This conclusion is supported by data comparing the expression level of C_i_ assimilation systems in *Synechococcus*, which is fully adapted to bicarbonate-containing media, to that in cells grown in ordinary BG-11 medium with no bubbling and a priori having a fully induced CCM (Figure S4). In this situation, we found similar levels of NDH-1_3_ expression in all variants, implying that cells equally sensed the low level of exogenous CO_2_. Simultaneously, cells that have been fully adapted to a bicarbonate-containing environment suppress both HCO_3_^−^ uptake systems—BCT1 and SbtA (*cmpA* and *sbtA* genes). This appears to be a “proper” reaction to large amounts of exogenous HCO_3_^−^. SbtA expression drops even at 10 mM NaHCO_3_, whereas BCT1 is only suppressed at 50 mM. The BCT1 of *S*. *elongatus* PCC 7942 has a *K*_0.5_ (HCO_3_^−^) value of around 15 µM [41]. The precise *K*_0.5_ (HCO_3_^−^) value of SbtA could not be determined [42]. Based on these findings, we can expect that SbtA has a higher affinity for bicarbonate than BCT1.

### 2.5. Contribution of External CA to Physiological Responses of Synechococcus under Conditions Where CO_2_ and HCO_3_^−^ Are Simultaneously Available to Cells

To better understand the impact of external CA on C_i_ photosynthetic assimilation, we conducted a one-time assessment of *Synechococcus* transcriptional responses to different [HCO_3_^−^]/[CO_2_] supply. For this purpose, wild-type and TF cells were cultivated under standard conditions (BG-11, pH 7.5, bubbling with 1.5% CO_2_) and then transferred to the following experimental settings:

1. BG-11, pH 7.5, no bubbling. Severe restriction on C_i_. Cells only have access to CO_2_, which diffuses into the medium from the air, as well as to HCO_3_^−^, which is generated from CO_2_ according to the Henderson–Hasselbach equation at pH 7.5;
2. BG-11, pH 7.5, bubbling with 0.04% CO_2_. These conditions, like those in option 1, correspond to cell growth at atmospheric CO_2_ levels. However, due to bubbling, the aquatic environment is actively saturated with the corresponding level of carbon dioxide;
3. BG-11, pH 9.5, 50 mM NaHCO_3_, no bubbling. Cells have access to a high concentration of HCO_3_^−^ in the environment; dissolved CO_2_, which diffuses into the medium from the air, is also available. Due to the high pH, additional CO_2_ cannot be generated from HCO_3_^−^according to [34];
4. BG-11, pH 9.5, 50 mM NaHCO_3_, bubbling with 0.04% CO_2_. Cells have access to a high amount of HCO_3_^−^ as well as to atmospheric CO_2_ level; the saturation of the medium with the latter is maintained by bubbling;
5. BG-11, pH 9.5, 50 mM NaHCO_3_, bubbling with 1.5% CO_2_. Cells have access to high amounts of both HCO_3_^−^ and CO_2_.

**Figure 10:**
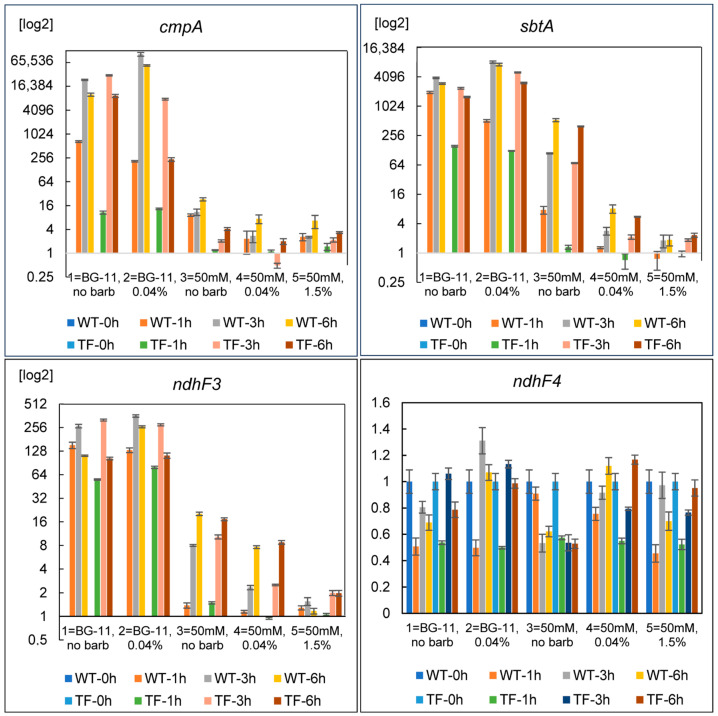
Changes in the relative normalized level of gene transcripts (Y axis) associated with C_i_ uptake systems in *Synechococcus* wild-type (WT) cells and that of transformant with constitutive expression of the L^Cya^-EcaA^Cya^ protein (TF) when transferred from standard cultivation conditions (BG-11, pH 7.5, 1.5% CO_2_) under conditions of different [HCO_3_^−^]/[CO_2_] supply (for more details, see the text). After collecting control samples, cells were pelleted by centrifugation and resuspended in experimental media. The level of gene expression is shown relative to that at the zero-hour point, corresponding to cell growth under standard conditions immediately before their transfer to experimental media. Data are normalized to the expression level of the *ppc* and *secA* genes.

After the transfer of the cells to the new environment, the expression levels of genes related to C_i_ assimilation systems were evaluated and compared to those under standard conditions (BG-11 media, pH 7.5, 1.5% CO_2_) (Figure 10). In all experimental variants, wild-type and TF cells exhibited similar responses. The only variation was in the strength of the effects that were observed: the TF often showed a less prominent transcriptional response when exposed to bicarbonate-containing media. This discrepancy was most likely caused by the TF’s outer CA’s capacity to “blur” the stress pattern by restoring the ratio of C_i_ forms in the pericellular region. Nonetheless, we found no significant phenotypic difference between wild-type and TF cells in the relevant experimental variants after three days of the experiment (Figure S5C). External CA activity appears to provide no discernible benefit to the TF under any of the experimental settings employed. In general, the obtained data confirmed the results of the prior experiment using bicarbonate media (Figure 9).

Due to the methodological changes, the results cannot be compared to those from the previous experiment aimed at lowering CO_2_ levels (Figure 5). The first experiment (Figure 5) involved immediate vessel changeover to barbotage with a gas–air combination containing less CO_2_. As a result, even after CO_2_ levels have decreased, certain crucial concentrations of C_i_ remain in the culture media. The current experiment (Figure 10) entailed changing the culture conditions by centrifuging the cells to remove them from the standard medium and then resuspending them in the experimental media. In this case, the cells were exposed to the novel conditions right away.

Variants 1 and 2 (BG-11, no bubbling or bubbling with 0.04% CO_2_) showed the greatest increase in the expression of the *cmpA*, *sbtA*, and *ndhF3* genes (Figure 10). On bicarbonate-containing media under 0.04% CO_2_ (variant No. 4), a significant increase in the expression of *cmpA*, *sbtA*, and *ndhF3* was observed only by the sixth hour after the cells were transferred to new conditions. While in settings with no bubbling (variant No. 3), the induction was visible as early as the third hour (Tables S1 and S2). In the first 6 h following transfer to new conditions, we did not notice significant changes in the mRNA level of the *ndhF4* gene (Figure 10).

These results may be interpreted as follows. Under normal growth conditions (BG-11, 1.5% CO_2_), *Synechococcus* does not experience photosynthesis-related C_i_ deficiency. CO_2_ entering the environment is in balance with HCO_3_^−^, which is generated from CO_2_ at pH 7.5 according to the Henderson-Hasselbach equation. The low-affinity CO_2_ uptake system (NDH-1_4_) appears to be responsible for C_i_ assimilation under these conditions. Furthermore, in an environment where CO_2_ is abundant, there is no need to maintain the energy-consuming HCO_3_^−^-uptake systems, BCT1 and SbtA.

When cells are switched from standard to CO_2_-limiting conditions (options No. 1 and No. 2: BG-11, without bubbling or bubbling with 0.04% CO_2_), they experience a simultaneous lack of both CO_2_ and HCO_3_^−^ due to a rapid decrease in total C_i_. That is why they activate all available C_i_-uptake systems, including NDH-1_3_, BCT1, and SbtA.

When cells are transferred to HCO_3_^−^-containing media, the rise in the expression of all inducible C_i_-uptake systems is modest for variant No. 5 (50 mM HCO_3_^−^ + 1.5% CO_2_), increases in variant No. 4 (50 mM HCO_3_^−^ + 0.04% CO_2_), and reaches a maximum value in variant No. 3 (50 mM HCO_3_^−^, no bubbling). Obviously, the induction of BCT1 and SbtA in variants 3–5 is related to the necessity to shift C_i_-assimilation from CO_2_ to HCO_3_^−^. The induction of NDH-1_3_ (with the exception of variant No. 5) is apparently associated with a decrease in CO_2_ amount in the medium at pH 9.5 as compared to pH 7.5. These results provide additional evidence that *Synechococcus* cells can sense a drop in the exogenous concentration of a specific type of C_i_ rather than the total concentration of CO_2_ and HCO_3_^−^ when they are shifted to new C_i_-supply conditions.

The strength of the induction of BCT1 and SbtA in “bicarbonate” variants Nos. 3–5 is inversely correlated with the level of CO_2_, which can be used as an extra source of exogenous C_i_ to HCO_3_^−^. This suggests that the possibility of energy-independent CO_2_ assimilation has a significant influence on the induction of HCO_3_^−^ uptake systems, even when this form of C_i_ is abundant. Variants Nos. 3–5 showed lower induction of NDH-1_3_, BCT1, and SbtA compared to Nos. 1–2, indicating the relevance of the total amount of available exogenous C_i_ (CO_2_ + HCO_3_^−^). Growth in optical density and dry biomass content in cell suspensions, as well as the overall look of the cultures in variants Nos. 1–5, shown in Figure S5A,B support these conclusions. Clearly, option 5 (50 mM NaHCO_3_ + 1.5% CO_2_) provides the optimal growth conditions.

These results may contribute to our understanding of the cell’s physiological processes in response to variations in the availability of various forms of C_i_. On the one hand, cyanobacteria can sense the total amount of exogenous C_i_ and adjust CCM activity in response to intracellular changes caused by variations in its availability. C_i_-limiting conditions alter cell biochemistry [8,39,43], resulting in increased intracellular levels of RBP and 2-phosphoglycolate, which are indicators of Calvin cycle repression and photorespiration activation, respectively. These molecules can function as effectors, modulating the ability of CCM-associated transcription factors to bind to DNA and regulate gene expression. At the post-translational level, CCM regulation may occur via adenyl nucleotides. Their ratio is directly related to the efficiency of photosynthesis, which in turn depends on the conditions of C_i_-supply [44].

On the other hand, the CCM operation can also be tuned based on the predominant form of exogenous C_i_. Thus, the cAMP molecule, which serves as a C_i_-sensing signal, triggers the regulation of the *sbtA* operon [45]. It has been demonstrated that the activity of soluble adenylate cyclase is directly proportional to the concentration of exogenous HCO_3_^−^ [46]. However, activation by CO_2_ has also been detected [47], implying that cAMP may play a role in the regulation of CO_2_-uptake system expression. The ability of allophycocyanin to bind CO_2_ may also indicate that it serves as a primary C_i_ sensor [48].

Our findings show that when the predominant form of exogenous C_i_ changes (without a simultaneous decrease in the total amount), *Synechococcus* experiences a lack of C_i_ entry into the cell, as evidenced by the induction of CCM components and the reorganization of the C_i_ uptake pattern based on its most accessible form. Consequently, in our case, we deal with the second variant of CCM regulation. The consistency of the molecular mechanisms underlying these processes remains to be elucidated.

### 2.6. Evaluation of the Appearance of Native External CA EcaA^Syn^ in Synechococcus under Different CO_2_/HCO_3_^−^-Supply Conditions

Previously, we demonstrated that *Synechococcus* cells lacked their own EcaA^Syn^ protein when cultured at 0.04 or 1.5% CO_2_ [11]. Here, we evaluated the emergence of EcaA^Syn^ in *Synechococcus* under a variety of conditions, including changes in CO_2_ and HCO_3_^−^ content, as well as their concentration ratios. Cells of both the wild-type and the TF were collected for the analysis, with the expectation that the latter would serve as a control variant: the presence of active EcaA^Cya^ would reduce the requirement for EcaA^Syn^ to appear. We evaluated the change in the level of *ecaA^Syn^* mRNA when cells were transferred from the standard (BG-11, 1.5% CO_2_) to experimental conditions and the presence of the corresponding protein product at the end of adaptation (6 h at 30 and 100% CO_2_ and 24 h for all other variants).

EcaA^Syn^ showed no transcriptional response under the majority of the conditions tested (Figure S6). However, in BG-11, in the absence of bubbling, cells exhibited an unexpectedly substantial rise in *ecaA^Syn^* expression 3 h after the onset of adaptation. Despite the observed oscillations, the protein product *ecaA^Syn^* was entirely missing in all experimental settings (Figure S7). Western blotting did not show any signal corresponding to the full-length (27 kDa) or processed (24.6 kDa) EcaA^Syn^ forms. It should be highlighted that, in addition to the sensitive signal visualization method, which detects femtogram levels of the protein, we utilized lengthy exposure times (up to 4.5 min), which would allow us to detect the presence of EcaA^Syn^ even in the smallest amounts. However, in all cases, the antibodies reacted nonspecifically with various polypeptides of *Synechococcus*. Thus, it was impossible to determine whether the EcaA^Syn^ protein has any physiological role.

### 2.7. Effect of Active External CA on Na^+^-Independent HCO_3_^−^ Uptake

*Synechococcus* cells can transport bicarbonate ions in either a Na^+^-independent or Na^+^-dependent manner. The former involves the ATP-driven BCT1 system, whereas the latter requires the SbtA symporter, which transports HCO_3_^−^ across the CM alongside the symport of Na^+^ ions and, hence, requires Na^+^-gradient to function [9].

As already mentioned in Section 2.4.2, the *K*_0.5_ (HCO_3_^−^) value for BCT1 of *S*. *elongatus* PCC 7942 is around 15 µM [41]. BCT1 provides a medium flux rate of HCO_3_^−^ into the cell. The exact *K*_0.5_ (HCO_3_^−^) value for SbtA (which has a low flux rate, at least in marine cyanobacteria [49]) was not determined [42]. Both BCT1 and SbtA are high-affinity systems, as they are activated only at low CO_2_ (Figure 5, Figure 6 and Figure 10).

Obviously, to assess the contribution of external CA to the Na^+^-independent consumption of HCO_3_^−^, it was necessary to work under conditions of low CO_2_ concentrations (0.04%) because, under optimal 1.5% CO_2_, cells satisfy the need for C_i_ primarily due to CO_2_ uptake using the low-affinity NDH-1_4_ system (Section 2.5).

**Figure 11:**
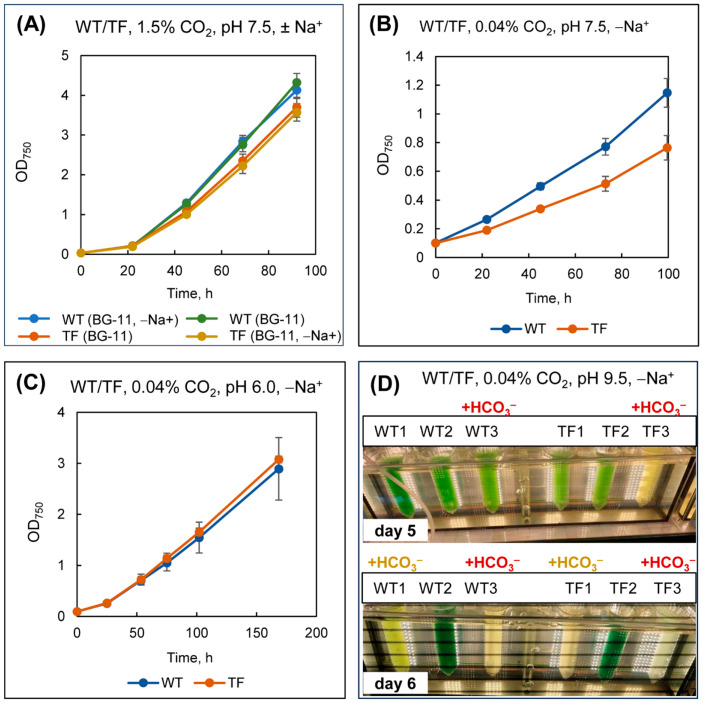
Effect of the presence of active external CA in *Synechococcus* on Na^+^-independent HCO_3_^−^ uptake. The increase in optical density of *Synechococcus* wild-type (WT) and transformant with constitutive expression of the L^Cya^-EcaA^Cya^ protein (TF) cell suspensions during cultivation is shown: (**A**) in standard BG-11 (pH 7.5) and Na^+^-depleted BG-11 (pH 7.5), under 1.5% CO_2_ bubbling; (**B**) in Na^+^-depleted BG-11 (pH 7.5) under 0.04% CO_2_ bubbling; (**C**) in Na^+^-depleted BG-11 (pH 6.0) under 0.04% CO_2_ bubbling. Panel (**D**) shows a general view of cultures growing in Na^+^-depleted BG-11 (pH 7.5) under 0.04% CO_2_ bubbling after the sequential addition of KHCO_3_ to individual vessels to a final concentration of 50 mM, which results in an increase in pH to 9.5.

Indeed, depletion in Na^+^ ions under optimal growth conditions (BG-11, pH 7.5, 1.5% CO_2_) had no noticeable effect on the growth of wild-type or TF cells (Figure 11A). In contrast, at low CO_2_ concentrations (0.04%) in a Na^+^-depleted medium (BG-11, pH 7.5), the TF grew substantially slower (Figure 11B). The growth rate of the cultures leveled off once more when they were cultivated on BG-11 minus Na^+^, pH 6.0 (Figure 11C). We explain these findings as follows. Because the ratio of equilibrium forms of C_i_ at pH 7.5 favors HCO_3_^−^, external CA activity will contribute to the HCO_3_^−^ predominance in the pericellular space of the TF. At the same time, the HCO_3_^−^ flow rate provided by Na^+^-independent BCT1 is insufficient for effective C_i_ uptake, as evidenced by the TF’s slow growth. In contrast to the TF, the CO_2_ substrate is still more easily accessible for wild-type cells. For this reason, under CO_2_-limiting conditions—that is, when the environment does not contain an oversupply of these molecules—the wild type can demonstrate its superiority. In a medium with a pH of less than 6.3, CO_2_ becomes the primary form of C_i_. Under these conditions, the presence of external CA activity will not exacerbate the depletion of Na^+^ ions because the CO_2_-uptake systems will still have access to CO_2_ in the pericellular space. This is why there is no difference between the cultivation of wild-type and TF cells in BG-11 minus Na^+^, pH 6.0.

After about four days of cultivation, KHCO_3_ at 50 mM was added to parts of the cultures growing in BG-11 minus Na^+^, pH 7.5, and 0.04% CO_2_, which further contrasted the differences between wild-type and TF cells. The TF dies within 24 h due to its inability to handle the circumstances, while the wild type still looks quite satisfactory (Figure 11D, upper panel, vessels WT3 and TF3). The addition of KHCO_3_ to other vessels (WT1 and TF1) leads, a day later, to a similar result (Figure 11D, lower panel). At this moment, the wild-type culture that received the first portion of KHCO_3_ (WT3) also dies one day after the TF1 transformant. The pH rises to 9.5 concurrently with the addition of KHCO_3_, and HCO_3_^−^ becomes the predominant form of C_i_ in the medium. It is evident that, even if the BCT1 system is active, it cannot ensure a significant supply of HCO_3_^−^ for photosynthesis. A vicious circle is created: BCT1 is powered by ATP energy; photosynthesis needs to be effective in order to generate ATP molecules in sufficient quantities; reduced C_i_ influx into the cell decreases the activity of the Calvin cycle followed by suppressing the light phase of photosynthesis, which is responsible for ATP synthesis.

These results lead to the following conclusion: under desalination conditions, with a decrease in the concentration of Na^+^ ions, even at somewhat alkaline pH, external CA activity in *Synechococcus* reduces C_i_ photosynthetic assimilation efficiency, thus giving a counter-advantage rather than a physiological priority.

### 2.8. The Impact of Active External CA on the Development of Oxidative Stress

At high light intensities, cyanobacteria may encounter oxidative stress due to an imbalance between the light and dark photosynthesis reactions, as well as the generation of ROS in cells. Low CO_2_ concentrations are predicted to exacerbate the situation by reducing the efficacy of the Calvin cycle. The latter scenario will be prevented once the CCM is active.

As previously mentioned, we observed the induction of expression of the own *ecaA^Syn^* gene in *Synechococcus* cells grown in BG-11 medium without bubbling with air or gas–air mixture (Figure S5B; variant No. 1). Since these severe C_i_-limiting conditions stimulate the formation of ROS, it can be expected that the attempt to trigger the mechanism of synthesis of the corresponding external CA may indicate its potential importance under these conditions.

ROS are formed in photosynthetic cells as singlet oxygen (^1^O_2_), superoxide anion (O_2_^−•^), hydroxyl radical (HO^•^), and hydrogen peroxide (H_2_O_2_) [50]. Hydrogen peroxide is the most stable ROS; therefore, adding H_2_O_2_ to the culture medium is widely used to simulate oxidative stress.

**Figure 12:**
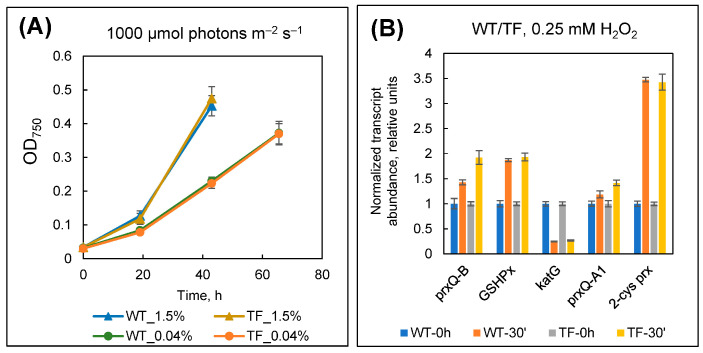
Assessing the role of external CA in the development of light and oxidative stress in *Synechococcus*. (**A**) Growth curves of *Synechococcus* wild-type (WT) and transformant with constitutive expression of the L^Cya^-EcaA^Cya^ protein (TF) under high light intensity (1000 μmol m^−2^ s^−1^) at 1.5% or 0.04% CO_2_ in gas–air mixture. The graphs represent standard deviations from the mean of three biological replicates. (**B**) mRNA levels of genes involved in hydrogen peroxide neutralization systems in wild-type and transformant cells 30 min after adding 0.25 mM H_2_O_2_. Gene designations are given in the text.

In this set of experiments, wild-type and TF cells were grown at 1.5% CO_2_ and low light intensity (30 µmol m^−2^ s^−1^ photons), then diluted to a low optical density (OD_750_ ~ 0.03), and then subjected to 1000 µmol photons m^−2^ s^−1^. The CO_2_ supply was constantly maintained at 1.5 or 0.04%. Because of their low optical density, the cells did not darken and were fully exposed to light stress. Cultivation occurred for the first 2–3 days, and the experiment was terminated when the suspensions reached an OD_750_ > 0.3. The evaluation of growth curves revealed no significant differences between the two types of cells, neither under optimal CO_2_ supply (1.5%) nor under CO_2_-limiting conditions (0.04%) (Figure 12A). The spectral properties of the cell cultures were likewise similar.

The intracellular system of ROS neutralization in *S*. *elongatus* PCC 7942 is far less studied than that of *Synechocystis* sp. PCC 6803. According to the literature [51,52] and Cyanobase database (http://genome.microbedb.jp/cyanobase/, accessed on 19 July 2024), *Synechococcus* cells contain the following potential components of the system for H_2_O_2_ neutralization: (1) catalase, encoded by the Synpcc7942_1656 gene (*katG*), (2) glutathione peroxidase (GSHPx, Synpcc7942_1214), (3) 1-Cys peroxiredoxin (*1*-*cys prx*, Synpcc7942_2449), (4) 2-Cys peroxiredoxin/thioredoxin peroxidase (*2*-*cys prx*, Synpcc7942_2309), (5) PrxQ-A1 peroxiredoxin/thioredoxin peroxidase (*prxQ*-*A1*, Synpcc7942_2180), (6) PrxQ-A2 peroxiredoxin (*prxQ*-*A2*, Synpcc7942_1806), (7) PrxQ-A3 peroxiredoxin/thioredoxin peroxidase (*prxQ*-*A3*, Synpcc7942_1942), (8) PrxQ-B peroxiredoxin/thioredoxin peroxidase (*prxQ*-*B*, SynPCC7942_0642).

For the analysis, five genes were selected (*katG*, *GSHPx*, *2*-*cys prx*, *prxQ*-*A1,* and *prxQ*-*B*) that, among other things, exhibited the strongest response to cell treatment with hydrogen peroxide, according to earlier data [51,52]. The assessment of changes in the expression levels of these genes after the addition of 0.25 mM H_2_O_2_ revealed no difference between wild-type and TF cells (Figure 12B). The data indicate that the injection of H_2_O_2_ causes comparable responses to oxidative stress in both cell types. Thus, the presence of active external CA appears to have no effect on cells’ resistance to this type of ROS.

## 3. Materials and Methods

### 3.1. Construction of Plasmids

All enzymes were purchased from Thermo Fisher Scientific (Vilnius, Lithuania), New England Biolabs (Ipswich, MA, USA), or Evrogen JSC (Moscow, Russia). Total nucleic acids from cyanobacteria were isolated using the phenol method [53], and RNA was removed using RNase A. Purified genomic DNA served as a template for PCR. Oligonucleotide primers (Table S3) with restriction endonuclease sites at their 5′ ends were synthesized by Evrogen JSC. DNA fragments were amplified with high-fidelity DNA polymerases.

Two DNA fragments were amplified from the *ecaA* gene of *Cyanothece* sp. ATCC 51142 (CyanoBase ID: cce_4328). One corresponded to the entire sequence of *ecaA^Cya^*, including the 81-bp starting region at the 5′ end that encodes the protein signal peptide (*L^Cya^*-*ecaA^Cya^*, 780 bp); the other represented a gene variation missing this region (*ecaA^Cya^*, 699 bp). Both PCR products had an extra 49 base pairs from the gene’s 3′ end. This feature was created to make it easier to select a reverse primer while keeping the gene’s natural stop codon.

The *ecaA* gene from *Synechococcus elongatus* PCC 7942 (CyanoBase ID: Synpcc7942_1388) was also amplified in two versions: (a) a full-length sequence (714 bp) including the region encoding the signal sequence (*L*^Syn^-*ecaA^Syn^*) and (b) a fragment corresponding to the mature protein (*ecaA^Syn^*, 648 bp).

The DNA fragment encoding the TorA protein’s signal peptide (*L^torA^*) was recovered using PCR from the genomic DNA of *Escherichia coli* strain BL21 (Novagen-Merck, San Diego, CA, USA). The *torA* gene, which encodes trimethylamine-*N*-oxide reductase, is highly conserved (up to 100%) across *E*. *coli* strains. Amplification primers were created using the *torA* sequence of *E*. *coli* strain K-12 (GenBank NC_000913.3).

The amplified segments were cloned in *E*. *coli* XL1-Blue cells (Agilent Technologies, La Jolla, CA, USA) using the pTZ57R vector (Thermo Fisher Scientific, Vilnius, Lithuania). The fragments were then digested at restriction sites on their ends and utilized to construct the following recombinant plasmids based on the pTrc99a vector (Pharmacia, Uppsala, Sweden) (Table S3):

1. pTrc99::*L^Syn^-ecaA^Syn^*. The DNA fragment *L*^Syn^-*ecaA^Syn^* was cloned into *Nco*I and *Bam*HI sites of pTrc99a;
2. pTrc99a::*L^torA^-ecaA^Syn^*. The DNA fragment *L^torA^* was cloned into *Nco*I and *Eco*RI sites of pTrc99a; then the construct pTrc99::*L^torA^* was treated with *Eco*RI and *Bam*HI restriction endonucleases and ligated to *ecaA^Syn^* fragment that possessed the analogous restriction sites at its ends;
3. pTrc99a::*L^Cya^-ecaA^Cya^*. The DNA fragment *L^Cya^-ecaA^Cya^* was cloned into *Nco*I and *Bam*HI sites of pTrc99a;
4. pTrc99a::*L^torA^-ecaA^Cya^*. The plasmid was assembled as described for variant 2 (pTrc99a::*L^torA^-ecaA^Syn^*). The *ecaA^Cya^*-fragment that possessed the *Eco*RI and *Bam*HI restriction sites at its ends was ligated with pTrc99::*L^torA^*, which was treated with analogous restriction endonucleases.

**Figure 13:**
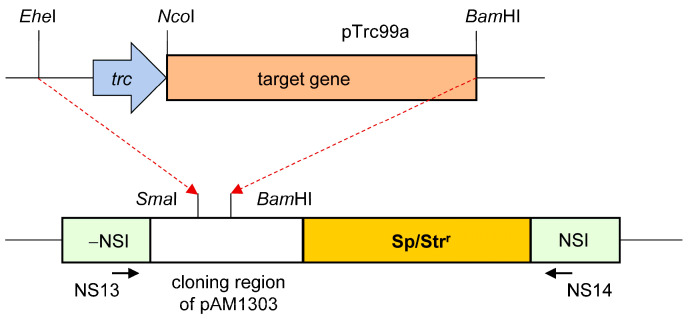
Scheme to generate constructs for *S. elongatus* PCC 7942 transformation using the pAM1303 vector. Sp/Str^r^: spectinomycin/streptomycin resistance cassette. NSI and -NSI are the neutral-site sequences that undergo double recombination with the *Synechococcus* chromosome. The figure also depicts the positions of primers NS13 (forward) and NS14 (reverse), which were then used to screen transformant colonies.

After obtaining constructs based on pTrc99a, the regions included the vector’s promoter (*trc*) region, and the subsequent region encoding the target protein was excised using *Ehe*I and *Bam*HI restriction endonucleases (Figure 13). The isolated segment was ligated into the pAM1303 vector [23] that was digested with *Sma*I and *Bam*HI restriction endonucleases. The resultant constructs were cloned in *E*. *coli* XL1-Blue cells in the presence of streptomycin, taking into account the strain’s spectinomycin resistance. The constructions were subsequently used to transform *S*. *elongatus* PCC 7942 cells. Sanger-type nucleotide sequencing (Evrogen) revealed that the observed structures were correctly assembled.

### 3.2. Transformation of Synechococcus

*S*. *elongatus* PCC 7942 cells were transformed with constructs based on the pAM1303 vector using the cyanobacterium’s natural competence [54]. Transformant colonies were selected on Petri dishes using BG-11 agar medium [55] and spectinomycin. The insertion of the target DNA sequence into a neutral site of the *Synechococcus* chromosome conditioned by the vector design [23] was detected using PCR. For screening, primers NS13 (5′-GTGCAGCAGCAACTTCAAG) and NS14 (5′-GTGCGTTCCACAGACATC) were employed [56] (Figure 13). The presence of specific mRNA encoding target recombinant proteins in *Synechococcus* transformant cells was determined using real-time or semi-quantitative PCR (Section 3.6).

### 3.3. Culture Conditions and Evaluation of Growth Parameters of Synechococcus

Standard conditions for culturing wild-type cells or transformants of *S*. *elongatus* PCC 7942 assumed photoautotrophic growth at 32 °C in BG-11 medium with the addition of a buffer agent (20 mM HEPES-NaOH, pH 7.5) under constant illumination with warm white LED lamps at an intensity of 100–150 μmol m^−2^ s^−1^ photons and bubbling with a gas–air mixture containing 1.5% CO_2_. Cultivation was performed in glass culture vessels using a laboratory-intensive culture system [57]. To isolate genomic DNA, *Cyanothece* sp. ATCC 51142 cells were cultivated in ASP2 medium under similar conditions.

Alternatively, in some experiments, cultivation was carried out in a Sanyo Versatile Environmental Test Chamber MLR-351 (Sanyo Electric Co., Osaka, Japan) in flat-bottomed flasks or penicillin vials capped on top with silicone or cellulose stoppers that allow gas exchange. In this scenario, the cultures were grown without bubbling, with occasional shaking, at 32 °C and illuminated with fluorescent lamps at 50 µmol m^−2^ s^−1^ photons.

Under experimental conditions, cultures were left without bubbling (only CO_2_ in the medium that presented in equilibrium with the surrounding air was available to them). Alternatively, bubbling was performed with air containing a natural atmospheric concentration of CO_2_ (0.04%) or with a gas–air mixture containing 10% (moderately high concentration), 30% (high concentration), and 100% (extremely high concentration) CO_2_. To explore the impact of light stress on growth, cells were preadapted to 30 µmol m^−2^ s^−1^ photons, which increased to up to 1000 µmol m^−2^ s^−1^ during the experiment.

The experiments employed the alternative variants of the BG-11 media listed below: (1) without Na_2_CO_3_ and HEPES-NaOH but containing NaHCO_3_ from 10 to 200 mM (pH ~ 9.5 for all options); (2) with Na^+^ ion depletion, replacing NaNO_3_ and Na_2_CO_3_ with equimolar amounts of KNO_3_ and K_2_CO_3_, respectively, and HEPES-NaOH with HEPES-KOH (pH 7.5). In a study to determine the influence of ambient acidification on the expression of genes encoding C_i_ uptake and Na^+^/H^+^ balance systems, 20 mM MES, pH 6.0, was utilized as a buffer agent [58]. In tests to imitate oxidative stress, H_2_O_2_ was added to the medium at a final concentration of 0.25 mM [59].

The media have been prepared using chemicals with a purity of at least 99.5% from the manufacturers listed below: Chimmed (Moscow, Russia), Servicebio (Wuhan, China), AppliChem (Darmstadt, Germany), Merck (Darmstadt, Germany), neoFroxx (Einhausen, Germany), CDH (New Delhi, India). Buffer agents were obtained from neoFroxx (HEPES, MES) or Merck (Trisma base). Solid media were prepared using bacteriologic agar-agar purchased from BD (Waltham, MA, USA) or Dia-M (Moscow, Russia). Water was purified by using a Milli-Q Plus system (Merck Millipore, Burlington, MA, USA). Hydrogen peroxide was obtained from Merck.

The optical density of cells was measured at 750 nm (OD_750_). Absorption spectra of cell suspensions were measured at wavelengths ranging from 350 to 750 nm. The measurements were taken using a Genesys 40 spectrophotometer (Thermo Fisher Scientific, Waltham, MA, USA).

The assessment of the amount of dry biomass in the volume of cell suspension was performed as described before [60].

### 3.4. Cell Fractionation, Electrophoresis and Immunoblotting

*Synechococcus* wild type or transformants cells were grown until OD_750_ value reached ~2. To analyze the appearance of *Synechococcus*’s own protein L^Syn^-EcaA^Syn^, we employed cells that had been acclimated to the experimental conditions for 24 h. Cell disruption and fractionation were carried out, as reported before [11]. A supernatant fraction rich in soluble proteins from the cytoplasm and periplasmic space was used for the studies. The protein content of the samples was determined using a commercial DC Protein Assay Kit (Bio-Rad Laboratories, Hercules, CA, USA).

Proteins were separated by electrophoresis in 12.5% denaturing PAGE [61]. Precision Plus Protein™ All Blue Standards (Bio-Rad) served as molecular weight markers. Gels were stained with Coomassie Brilliant Blue R-250.

Western blotting was carried out according to Bio-Rad Laboratories protocols. Proteins have been transferred onto a nitrocellulose membrane. The primary antibodies used were as follows: (1) Rabbit polyclonal antibodies against the EcaA^Cya^ protein of *Cyanothece* sp. ATCC 51142 [6]; (2) Rabbit polyclonal antibodies against the EcaA^Syn^ protein of *S*. *elongatus* PCC 7942 [11]. To assess signal specificity when utilizing anti-EcaA^Syn^, antibodies were preincubated with the excess of the recombinant EcaA^Syn^, as described in [11]. Antibodies against rabbit immunoglobulins fused to horseradish peroxidase were used as secondary antibodies (Cytiva, Marlborough, MA, USA; NA934). Antibody-antigen complexes were visualized with ClarityTM Western ECL substrates (Bio-Rad Laboratories, Hercules, CA, USA). Signals were detected using a ChemiDoc MP system and Image Lab 5.1 software from Bio-Rad.

### 3.5. Assay of Carbonic Anhydrase Activity

*Synechococcus* cells, both wild-type and transformants, were grown until OD_750_ ~ 2, then collected by centrifugation (3500× *g*, 10 min, 4 °C), washed with chilled 30 mM HEPES-KOH buffer (pH 8.2), and suspended in the same buffer. CA activity was assessed electrometrically [62] by monitoring the rate of H^+^ evaluation during CO_2_ hydration, as previously described [6,11]. pH measurements were taken every 0.5 s. The specificity of the reactions was validated by pre-incubating the samples for 30 min with the CA inhibitor ethoxyzolamide at a final concentration of 0.5 mM. The CA activity was expressed in Wilbur-Anderson units (WAU) per 1 mg of total cell protein.

### 3.6. Samples Collection, RNA Isolation, RT-qPCR and Semi-Quantitative RT-PCR

To determine the existence of specific mRNA in *Synechococcus* transformants with constitutive expression of various external CAs, cells grown under standard conditions were employed. Wild-type cells served as the control.

In experiments to investigate the role of active external CA in *Synechococcus* physiology, cultures of wild-type cells (WT) and the transformant constitutively expressing the L^Cya^-EcaA^Cya^ protein (TF) were cultivated under standard conditions before being collected as control samples. To investigate the transcriptional response of cells during adaptation to different CO_2_ concentrations, vessels containing culture suspensions were directly transferred to bubbling with a gas–air mixture with varying carbon dioxide content. All other growth conditions were unchanged. Culture samples for RNA isolation (25 mL of culture suspensions at OD_750_ ~ 1) were collected at specific time intervals after the beginning of adaptation, according to [53].

In experiments involving culture medium replacement, cells were pelleted by centrifugation (3500× *g*, 10 min, at room temperature) after the collection of control samples, rinsed with the experimental medium, and resuspended in it. The cultures were then returned to the previous growth conditions or subjected to additional changes in the concentration of exogenous CO_2_. Samples were withdrawn in the same manner as in the previous case.

In oxidative stress modeling experiments, cultures were grown under standard conditions, control samples were taken, and then H_2_O_2_ was added to the medium at a final concentration of 0.25 mM [59]. Samples were collected 30 min after the onset of the treatment.

Each experiment was performed on three biological replicates. The selected cell samples were averaged from each variety. Each experiment was repeated at least three times independently. Section 2 presents typical data from biological replicates.

Total RNA was isolated from *Synechococcus* cells as described earlier [53] and then additionally purified with DNase I (Thermo Fisher Scientific, Vilnius, Lithuania). The manufacturer’s protocol was followed for cDNA synthesis with MMLV reverse transcriptase (Evrogen, Moscow, Russia) and random decanucleotide primers (Evrogen).

For RT-qPCR, the supermix qPCRmix-HS SYBR (Evrogen) was used. The selection of gene-specific primers was performed as previously reported [11] or using literature data [29,63]. Synthetic oligonucleotides used as primers were synthesized by Evrogen (Table S4). The reaction was performed in the CFX96 Touch™ Real-Time PCR Detection System with Image Lab 5.1 software (Bio-Rad). The standard cDNA amplification process for 40 cycles included 3 min of pre-denaturation at 95 °C, 30 s of denaturation at 95 °C, 30 s of annealing at 54 °C, 30 s of extension at 72 °C, and melting curve analysis. The reaction was carried out in three technical replicates for each sample/gene pair. The data were calculated using the CFX Manager 3.1 software tool (Bio-Rad) using the ΔΔC_T_ method. The data were normalized to the transcript levels of the *secA* (CyanoBase, Synpcc7942_0289), *petB* (Synpcc7942_2331), *ilvD* (Synpcc7942_0626), and *ppc* (Synpcc7942_2252) genes, which were pre-selected as maintaining expression stability (change in transcript level less than two-fold) under the experimental conditions used.

Semi-quantitative RT-PCR was performed using the same primers as for RT-qPCR (Table S4). PCR was carried out using the Hot-start Taq DNA polymerase (Evrogen). The amplification technique for 25 cycles included 3 min of pre-denaturation at 95 °C, 30 s of denaturation at 95 °C, 20 s of annealing at 58 °C, 30 s of extension at 72 °C, and 3 min of final incubation at 72 °C.

### 3.7. Data Visualization

Presentation of graphical content was carried out using the software MS Excel 2019 MSO. The amino acid alignment (Supplementary Materials, Figure S8) was performed using the Clustal V algorithm of the MegAlign module of Lasergene v. 12.3.1 software package (DNAStar Inc., Madison, WI, USA). All Figures were prepared using MS PowerPoint 2019 MSO.

## 4. Conclusions

The external α-CA EcaASyn from *S*. *elongatus* PCC 7942 has not yet been shown to have a clear physiological role in standard laboratory culture conditions. In this study, we assess the appearance of EcaA^Syn^ in *Synechococcus* under a wide range of experimental conditions, varying in the level and ratio of CO_2_ and HCO_3_^−^ concentrations, which could not confirm the presence of the protein in the cells, despite some fluctuations in the amount of the corresponding transcript (Figures S6 and S7).

Furthermore, a number of facts imply that *Synechococcus*’ intracellular mechanism is specifically targeted at preventing the appearance of EcaA^Syn^. Thus, constitutive expression of various types of external CAs in *Synechococcus* cells (Table 1) was successful only when these proteins were different from their own EcaA^Syn^ (Figure 1, Figure 2 and Figure 3 and Table 2). It seems that *Synechococcus* recognizes and destroys both transcripts and protein products only for its own external CA. However, it is unable to detect the appearance of homologous nucleotide sequences or proteins.

Previously, we found that when L^Syn^-EcaA^Syn^ was heterologously expressed in *E*. *coli*, the recombinant full-length protein remained within cells [11] that exhibited no external CA activity. This observation indicates that the relevant bacterial Tat export system does not recognize the protein signal peptide. This may happen due to a loss of phenylalanine residue required for Tat recognition (Figure S8). These results obtained for *E*. *coli* can also be generalized to *Synechococcus* cells, as both of these species are gram-negative prokaryotes with similar protein export mechanisms for translocation through the CM. It appears that even if L^Syn^-EcaA^Syn^ was effectively translated in the *Synechococcus* cells, the protein would be unable to pass through the CM and enter the periplasm.

To summarize, the appearance of EcaA^Syn^ in *Synechococcus* periplasm is constrained at several stages: low mRNA levels, protein digestion by proteases, and issues with secretion through CM. From these perspectives, comparing the physiology of wild-type *Synechococcus* cells to their transformant with an artificially inserted active external CA was intriguing.

In this study, we created a number of *Synechococcus* transformants with constitutive expression of extracellular CAs (Table 1). One of them with full-length EcaA protein from *Cyanothece* sp. ATCC 51142 (L^Cya^-EcaA^Cya^) was selected, as it showed the most prominent feature of external CA activity while lacking the recombinant protein in the cytoplasm. This transformant was designated here as “TF”. To assess the role of an “additional” external CA in *Synechococcus* physiology, a variety of investigations were carried out in which conditions were simulated to mimic the oscillations that occur in the natural environment of this cyanobacterium.

Most of the experiments revealed no substantial differences between wild-type and transformant cells. This assertion pertains to culture growth and physiological parameters across various CO_2_ and HCO_3_^−^ concentrations and their ratios (Figure 4, Figures S2, S3 and S5), as well as to adaptive cell reactions to changes in cultivation modes (Figure 6, Figure 7, Figure 8, Figure 9 and Figure 10). Simultaneously, when the level of exogenous CO_2_ was drastically decreased (from 1.5 to 0.04%), TF cells demonstrated a disadvantage compared to the wild type. From the data obtained (Figure 5), we concluded that the active external CA of the transformant contributed to a more rapid removal of CO_2_ from the medium. Therefore, TF cells occurred under C_i_-limiting conditions earlier compared to wild-type cells.

A similar conclusion indicating a counter-advantage of external CA rather than a physiological priority was achieved when analyzing the contribution of EcaA^Cya^ to Na^+^-independent HCO_3_^−^ consumption (Figure 11). Under desalination, when the concentration of Na^+^ ions decreases, external CA activity reduces the efficiency of photosynthetic C_i_ assimilation, which is especially true in alkaline environments.

Experiments involving the adaptation and cultivation of *Synechococcus* at high and extremely high CO_2_ concentrations (30 and 100%) revealed no difference between the growth characteristics and transcriptional response of both cell types (Figure 4 and Figure 6). Thus, these data do not support our hypothesis regarding the protective role of external CAs in the conditions of an ancient CO_2_-rich atmosphere [21]. Yet, these experiments showed for the first time that the NDH-1_4_ CO_2_ uptake system, previously thought to be constitutive, is gradually repressed by the increase of exogenous CO_2_ from natural (0.04%) to extremely high (100%) levels.

An examination of the influence of active external CA in *Synechococcus* on the development of oxidative and light stress revealed no differences between physiological parameters on the transcriptional response of the H_2_O_2_ neutralization systems in wild-type and transformant cells (Figure 12). It indicates that the presence of periplasmic CA has no influence on cell resistance to these ROS.

Based on the results of our research on *Synechococcus* adaptation to fluctuating [CO_2_]/[HCO_3_^−^] supply (Figure 9, Figure 10 and Figure S5), we made conclusions that contribute to our understanding of the cell’s physiological processes in response to variations in the availability of various forms of C_i_. Our findings show that when the predominant form of exogenous C_i_ changes (without a simultaneous decrease in the total amount), *Synechococcus* experiences a lack of C_i_ entry into the cell, as evidenced by the induction of CCM components and the reorganization of the C_i_ consumption pattern based on its most accessible form. Simultaneously, the possibility of energy-independent assimilation of CO_2_ has a significant influence on the strength of the induction of HCO_3_^−^ uptake systems, even when HCO_3_^−^ form of C_i_ is abundant. These observations suggest that the predominant form of exogenous C_i_ can serve as a primary signal for the reconstruction of the CCM architecture. The consistency of the molecular mechanisms underlying these processes remains to be elucidated.

Summarizing the study, we conclude that *Synechococcus* does not normally require the presence of an active external CA. It is possible that the enzyme may have played a physiological role in *Synechococcus* at a certain evolutionary period. However, its uselessness in modern *Synechococcus* resulted in a reduction of the mechanisms that assure the appearance of active EcaA^Syn^ in the periplasmic space despite the preservation of the corresponding gene in the genome.

## Figures and Tables

**Table 1:** External CA proteins for the constitutive expression in *S. elongatus* PCC 7942.

Protein	Description	Export System	Reference
L^Syn^-EcaA^Syn^	Full-length EcaA of *Synechococcus* with its own native signal peptide that cannot ensure protein transfer across the CM	Tat	[11]
L^TorA^-EcaA^Syn^	EcaA protein of *Synechococcus* fused with the signal peptide of *E. coli* TorA * protein, with confirmed efficient transfer across the CM	Tat	[11,17]
L^Cya^-EcaA^Cya^	Full-length EcaA of *Cyanothece* sp. ATCC 51142 with its own functional signal peptide **	Sec	[6,13]
L^TorA^-EcaA^Cya^	EcaA from *Cyanothece* fused with the TorA signal peptide, allowing protein transfer through the CM	Tat	[13,17]

* trimethylamine *N*-oxide reductase A; ** the corresponding transformant was designated as “TF”.

**Table 2:** CA activity of intact wild-type *Synechococcus* cells as well as transformants with expression of different external CAs.

Synechococcus Cell Type	CA Activity, WAU/mg Total Cell Protein
WT	0
+ L^Syn^-EcaA^Syn^	0
+ L^TorA^-EcaA^Syn^	7.9 ± 0.3
+ L^Cya^-EcaA^Cya^ (TF)	178.1 ± 2.5
+ L^TorA^-EcaA^Cya^	80.8 ± 1.7

WAU values are calculated taking into account the total amount of protein used in the reaction. Standard deviations from 3–5 technical repetitions are shown.

## Data Availability

Data are contained within the article and Supplementary Materials.

## Supplementary Materials

The following supporting information can be downloaded at https://www.mdpi.com/article/10.3390/plants13162323/s1, Figure S1: PCR genotyping of *Synechococcus* transformant clones of different lines; Figure S2: Growth of *Synechococcus* wild-type and transformant TF (+L^Cya^-EcaA^Cya^) on standard BG-11 medium and on BG-11 with different NaHCO_3_ concentrations; Figure S3: A general view of *Synechococcus* wild-type and transformant TF (+L^Cya^-EcaA^Cya^) cells cultures on BG-11 medium with varying NaHCO_3_ concentrations; Figure S4: Comparison of the mRNA levels of genes associated with C_i_ uptake systems in *Synechococcus* wild-type and transformant TF (+L^Cya^-EcaA^Cya^) cells, fully adapted to growth on standard BG-11 medium, without bubbling or growing on BG-11 with the addition of NaHCO_3_ (10 or 50 mM); Figure S5: Growth of *Synechococcus* wild-type and transformant TF (+L^Cya^-EcaA^Cya^) under conditions differing in the content and ratio of HCO_3_^−^ and CO_2_; Figure S6: The level of transcripts of chromosomal copy of the own *ecaA^Syn^* gene in *Synechococcus* wild-type and transformant TF (+L^Cya^-EcaA^Cya^) cells under varied CO_2_/HCO_3_^−^ supply; Figure S7: Immunolocalization of EcaA^Syn^ in the soluble protein fraction of *Synechococcus* cells adapted to different CO_2_ or [HCO_3_^−^]/[CO_2_] supply compared to the standard conditions (1.5% CO_2_); Figure S8: The difference in a structure of the *n*-region of EcaA^Syn^ signal peptide between *S. elongatus* PCC 7942 and the classical signal sequences for transfer through the CM via the Tat translocation pathway; Table S1: Changes in mRNA levels of genes associated with C_i_ uptake systems in experimental variants 3 h after transferring cells from standard (BG-11, 1.5% CO_2_) to experimental growth conditions; Table S2: Changes in mRNA levels of genes associated with C_i_ uptake systems in experimental variants 6 h after transferring cells from standard (BG-11, 1.5% CO_2_) to experimental growth conditions; Table S3: Assembling of genetic constructs based on pTrc99a vector; Table S4: Nucleotide sequences of the synthetic oligonucleotides used as primers for RT-qPCR and semi-quantitative RT-PCR. References [64,65] are cited in the Supplementary Materials.
