# Supplementary material for: The Freshwater Cyanobacterium Synechococcus elongatus PCC 7942 Does Not Require an Active External Carbonic Anhydrase
Source: Plants (Basel). 2024 Aug 20;13(16):2323. doi: 10.3390/plants13162323 (PMC11360081; doi:10.3390/plants13162323)
Supplement: Supplementary file 1 [file plants-13-02323-s001.zip › Supplementary Figures_Kupriyanova_R1.pdf]

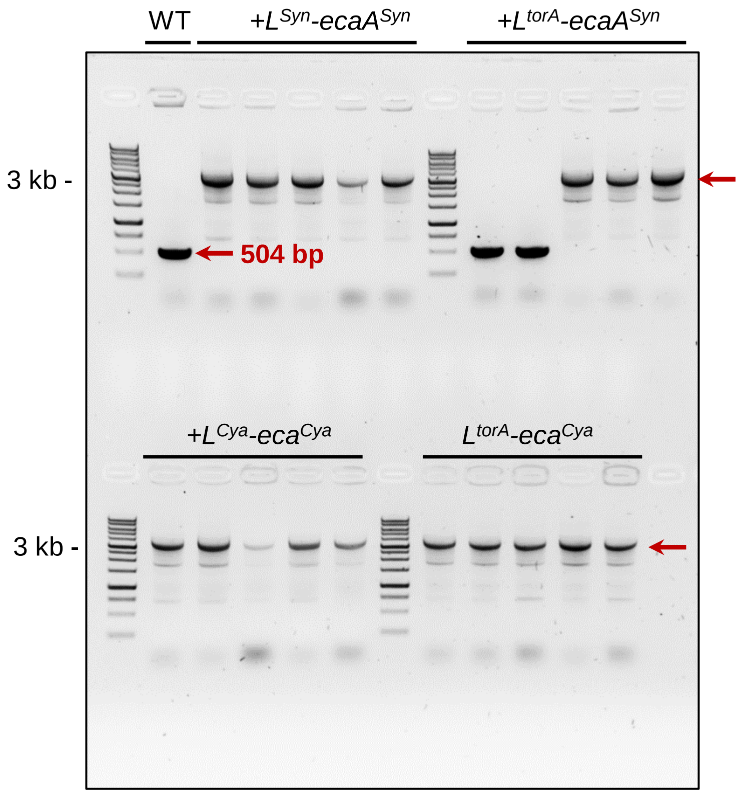

Figure S1. PCR genotyping of *Synechococcus* transformant clones of different lines. Genomic DNA of wild-type (WT) cells, as well as genomic DNAs isolated from transformants, were used as templates. Reactions were performed using the primer pair NS13 and NS14 (Wang et al. 2021; see also Figure 13 in the main text). The figure shows a negative image of an agarose gel stained with ethidium bromide after separation of PCR products. The size of the "wild-type" PCR fragment is 504 bp. Integration of the target DNA fragment into the genome resulted in the appearance of PCR fragments ranging in length from 3705 to 3807 bps in different transformant lines.

References to Figure S1:

Wang, B.; Xu, Y.; Wang, X.; Yuan, J.S.; Johnson, C.H.; Young, J.D.; Yu, J. A guanidine-degrading enzyme controls genomic stability of ethylene-producing cyanobacteria. *Nat. Commun.* **2021**, *12*, 5150. doi: 10.1038/s41467-021-25369-x

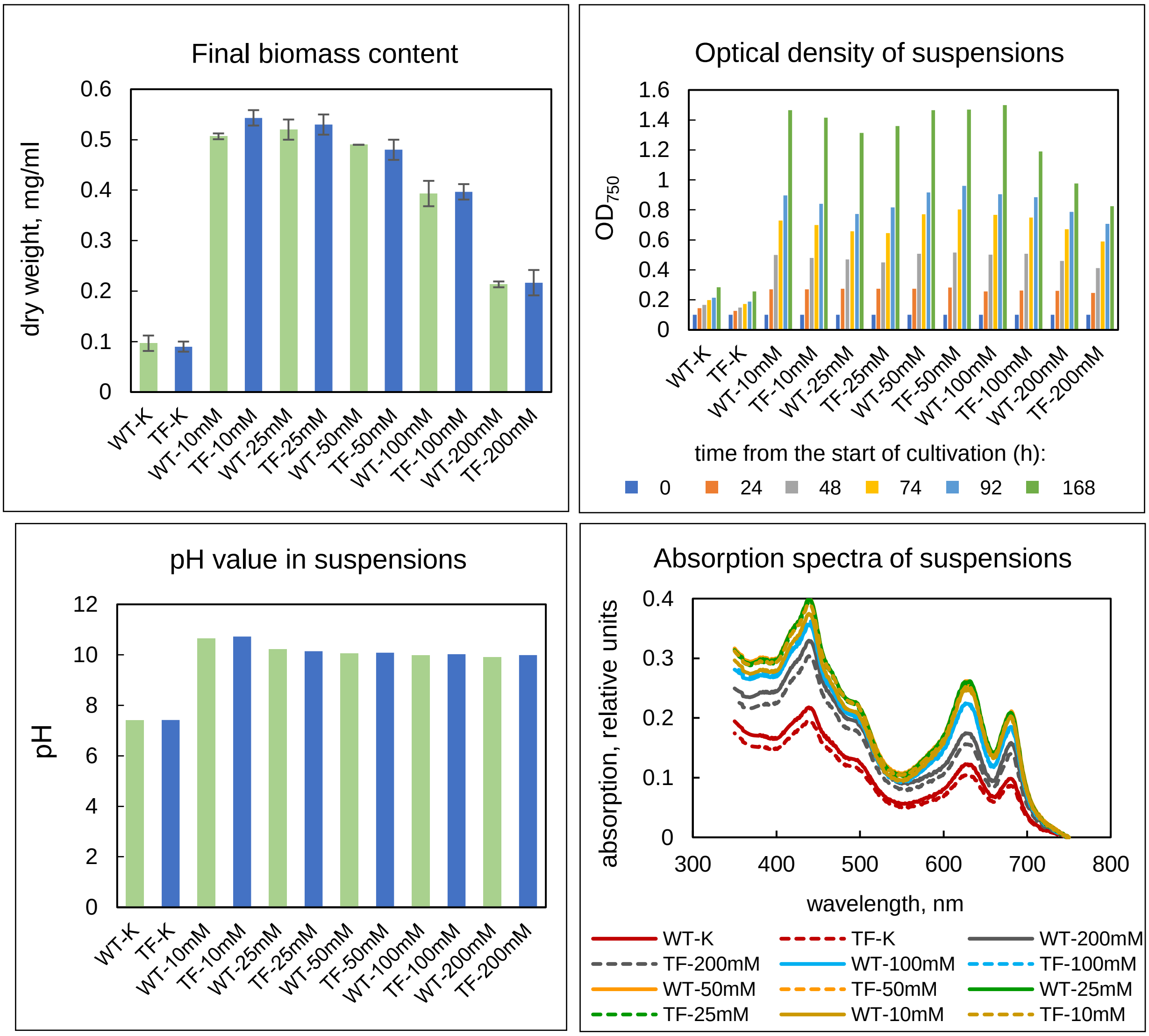

Figure S2. Growth of *Synechococcus* wild-type (WT) and transformant with constitutive expression of the L^{Cya}-$EcaA^{Cya}$ protein (TF) on standard BG-11 medium (control, K) and on BG-11 with different $NaHCO_3$ concentrations (from 10 to 200 mM). Dense cultures grown under standard conditions were diluted in experimental media to an initial optical density at 750 nm (OD_{750}) of 0.1. Cells were grown in flasks or penicillin vials, without additional bubbling, with periodic shaking, at 32 °C and illuminated with fluorescent lamps with an intensity of ~50 µmol m^{-2} s^{-1} photons. Cultivation was carried out for a week with periodic assessment of OD_{750}. At the end of cultivation, the biomass yield (dry weight of cells per unit volume of cell suspension) was determined. The pH of cultural suspensions and their absorption spectra were assessed in the middle of the cultivation cycle. The absorption spectra are aligned with that obtained at 750 nm.

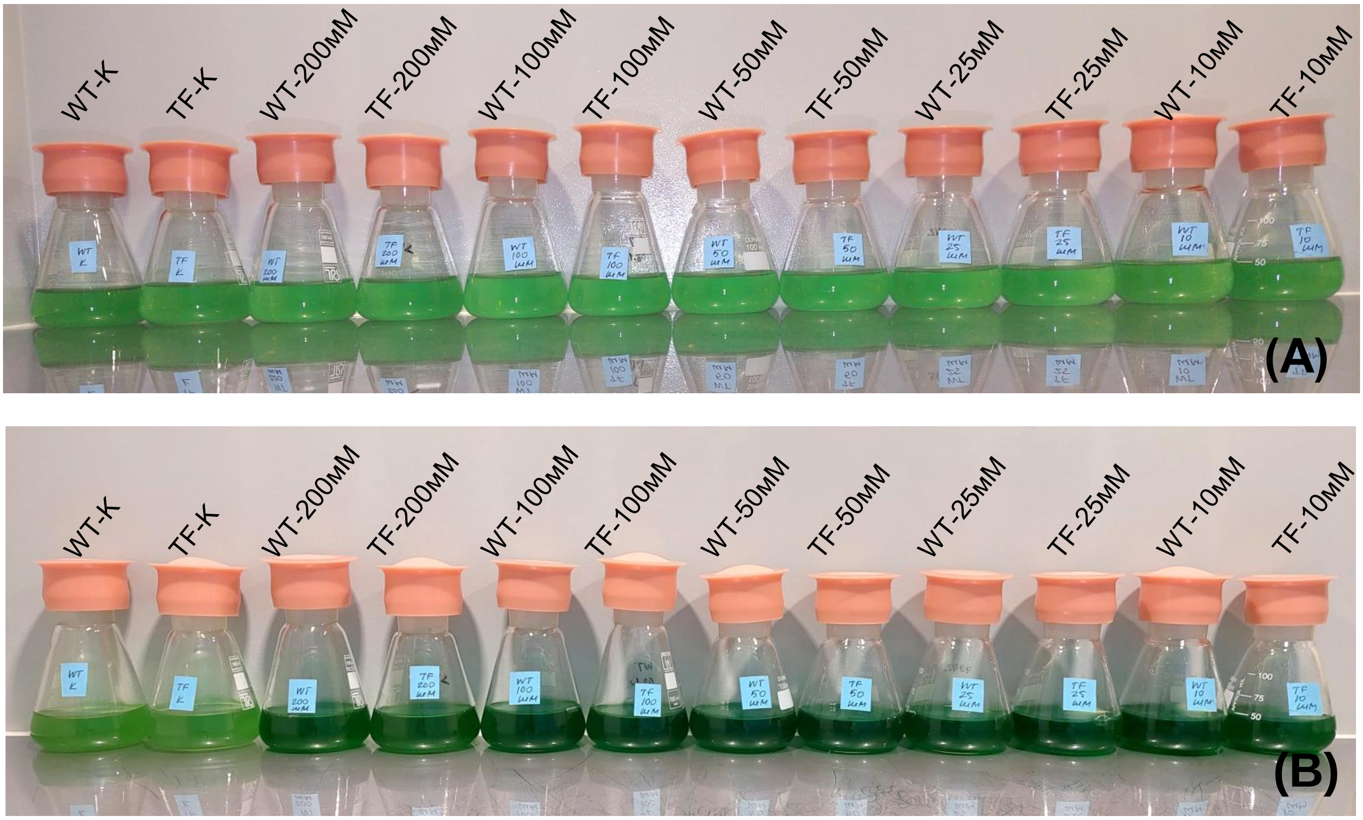

Figure S3. A general view of *Synechococcus* wild-type (WT) and transformant with constitutive expression of the L^{Cya}-$EcaA^{Cya}$ protein (TF) cells cultures on BG-11 medium with varying $NaHCO_3$ concentrations. (A) Start of experiment: immediately after dilution of the cultures to an identical starting optical density (OD_{750}) of 0.1; (B) On the fourth day of incubation.

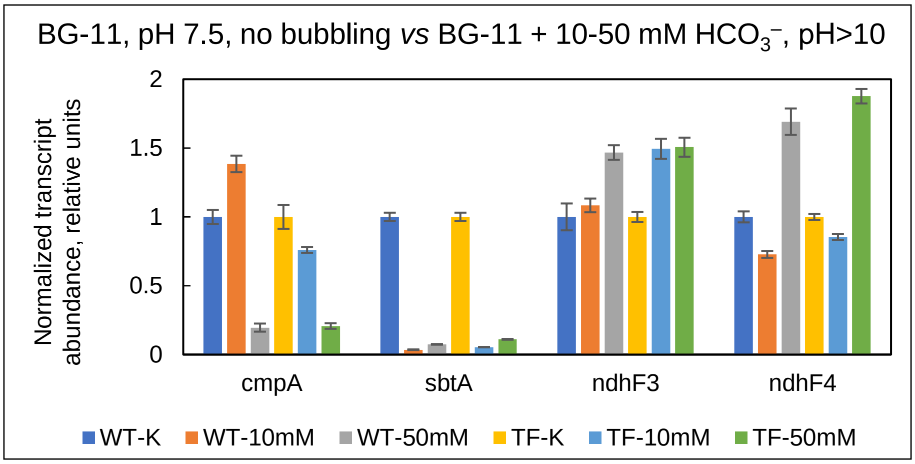

Figure S4. Comparison of the mRNA levels of genes associated with C_i uptake systems in *Synechococcus* wild-type (WT) cells and that of transformant with constitutive expression of the L^{Cya}-$EcaA^{Cya}$ protein (TF), fully adapted (3 days of incubation) to growth in standard BG-11 medium, pH 7.5, without bubbling (control variants, K) or growing on BG-11 with the addition of $NaHCO_3$ (10 or 50 mM, pH>10). The level of genes expression in cultures on bicarbonate media is shown relative to control cells. Data are normalized to the expression levels of the *ivlD* and *secA* genes.

(A)

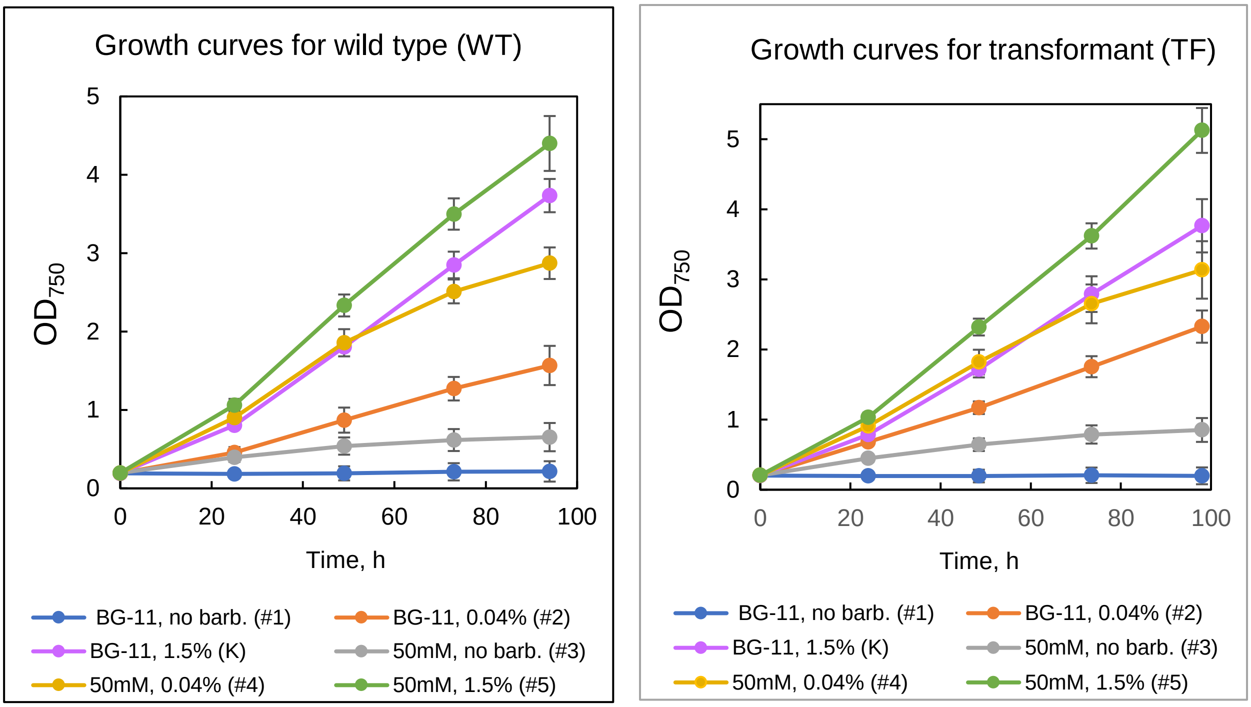
Growth curves for wild type (WT)
OD_{750}
5
4
3
2
1
0
0
20
40
60
80
100
Time, h
BG-11, no barb. (#1)
BG-11, 0.04% (#2)
BG-11, 1.5% (K)
50mM, no barb. (#3)
50mM, 0.04% (#4)
50mM, 1.5% (#5)
Growth curves for transformant (TF)
OD_{750}
5
4
3
2
1
0
0
20
40
60
80
100
Time, h
BG-11, no barb. (#1)
BG-11, 0.04% (#2)
BG-11, 1.5% (K)
50mM, no barb. (#3)
50mM, 0.04% (#4)
50mM, 1.5% (#5)

(B)

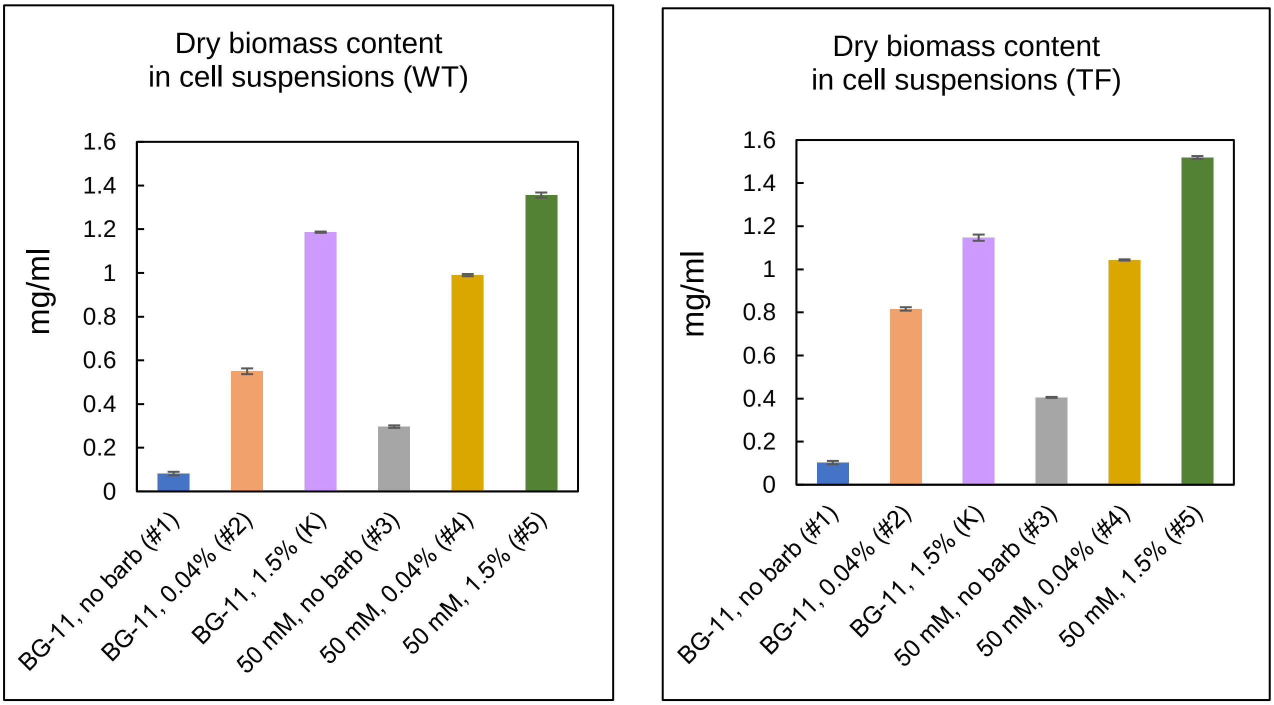
Dry biomass content
in cell suspensions (WT)
mg/ml
1.6
1.4
1.2
1
0.8
0.6
0.4
0.2
0
BG-11, no barb (#1)
BG-11, 0.04% (#2)
BG-11, 1.5% (K)
50 mM, no barb (#3)
50 mM, 0.04% (#4)
50 mM, 1.5% (#5)
Dry biomass content
in cell suspensions (TF)
mg/ml
1.6
1.4
1.2
1
0.8
0.6
0.4
0.2
0
BG-11, no barb (#1)
BG-11, 0.04% (#2)
BG-11, 1.5% (K)
50 mM, no barb (#3)
50 mM, 0.04% (#4)
50 mM, 1.5% (#5)

(C)

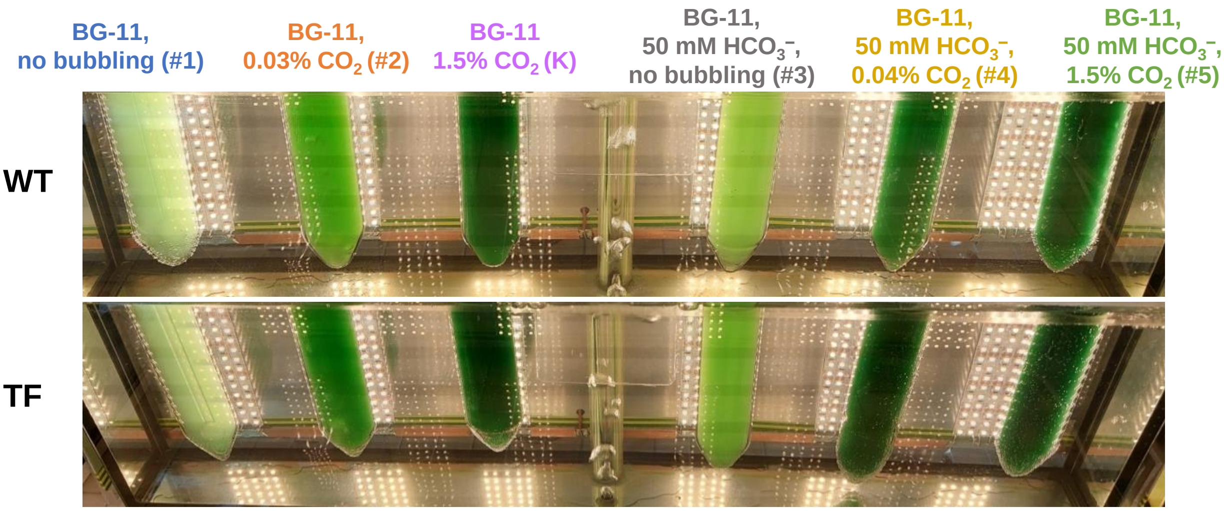

Figure S5. Growth of *Synechococcus* wild-type (WT) and transformant with constitutive expression of the L^{Cya}-$EcaA^{Cya}$ protein (TF) under conditions differing in the content and ratio of HCO_3^- and CO_2.. (A) Increase in optical density of cell suspensions. The graphic displays typical data from a single experiment run. (B) Content of dry biomass in cell suspensions at the end point of cultivation. (C) A general view of cultures on the third day after the start of cultivation. “K” is the control variant that corresponds to standard conditions (BG-11, pH 7.5, 1.5% CO_2). Cultivation conditions Nos. 1–5: 1 – BG-11, pH 7.5, without bubbling; 2 – BG-11, pH 7.5, 0.04% CO_2; 3 – BG-11 with 50 мM HCO_3^-, pH 9.5, without bubbling; 4 – BG-11 with 50 мM HCO_3^-, pH 9.5, 0.04% CO_2; 5 – BG-11 with 50 мM HCO_3^-, pH 9.5, 1.5% CO_2.

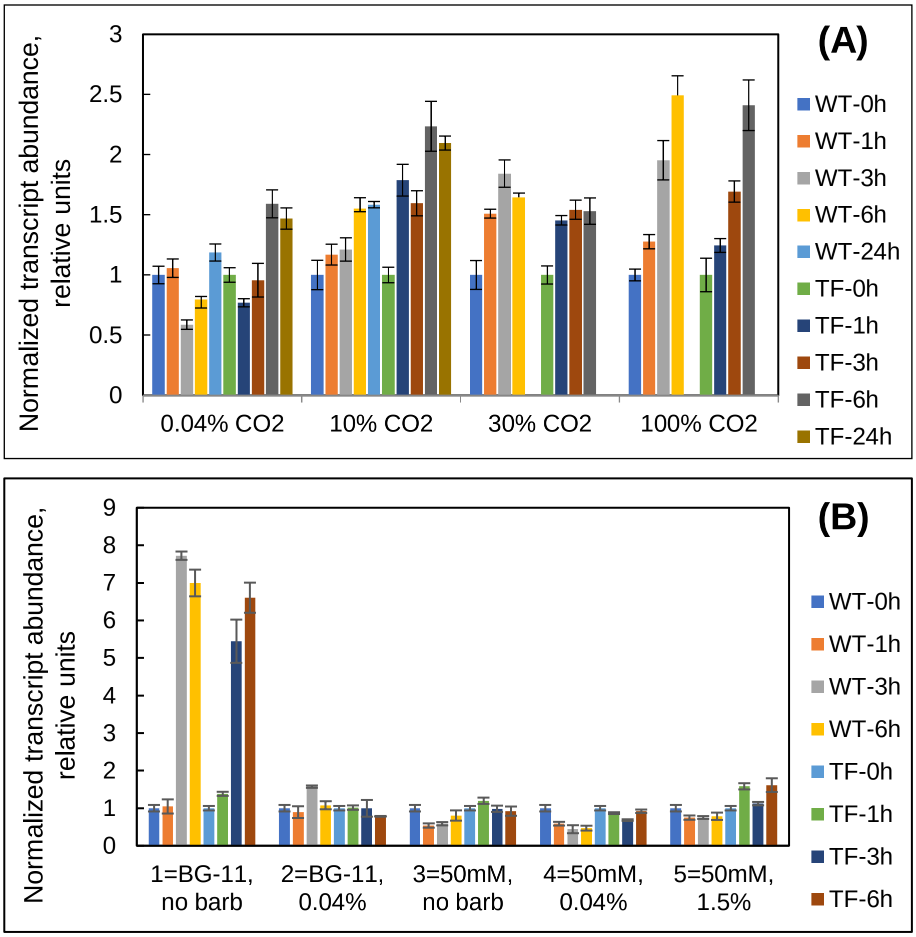

Figure S6. The level of transcripts of chromosomal copy of the own *ecaA*^{Syn} gene in *Synechococcus* wild-type (WT) cells and that of transformant with constitutive expression of the L^{Cya}-EcaA^{Cya} protein (TF) under varied CO_2/HCO_3^- supply. (A) When the CO_2 concentration in the gas-air mixture changed from 1.5% (point 0 h) to 0.04, 10, 30, or 100%. Data has been normalized to the expression levels of the *petB*, *secA*, *secA+ilvD*, or *ilvD* genes, respectively, for 0.04, 10, 30, and 100% CO_2. (B) When transferred from optimum conditions (BG-11, pH 7.5, 1.5% CO_2; point 0 h) under varied $[CO_2]/[HCO_3^-]$-supply (1 – BG-11, pH 7.5, without bubbling; 2 – BG-11, pH 7.5, 0.04% CO_2; 3 – BG-11 with 50 mM HCO_3^-, pH 9.5, without bubbling; 4 – BG-11 with 50 mM HCO_3^-, pH 9.5, 0.04% CO_2; 5 – BG-11 with 50 mM HCO_3^-, pH 9.5, 1.5% CO_2). The data has been normalized for *ppc* and *secA* expression levels. Transcript abundance in both panels is compared to that at 0 h.

(A)

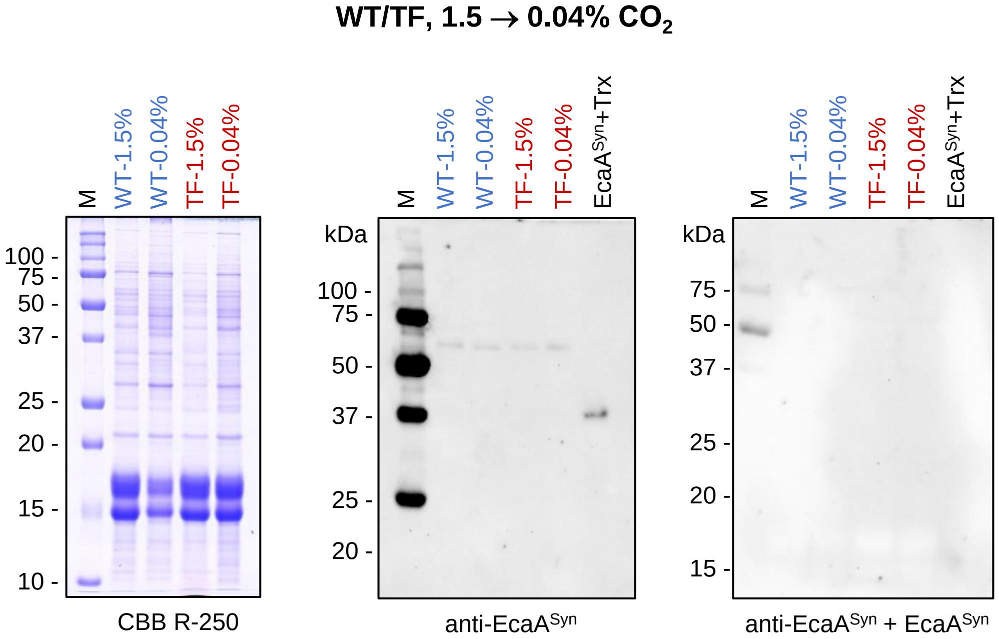
WT/TF, 1.5 → 0.04% CO_2
M
WT-1.5%
WT-0.04%
TF-1.5%
TF-0.04%
EcaA^{Syn}+Trx
kDa
100 -
75 -
50 -
37 -
25 -
20 -
15 -
10 -
CBB R-250
anti-EcaA^{Syn}
anti-EcaA^{Syn} + EcaA^{Syn}

(B)

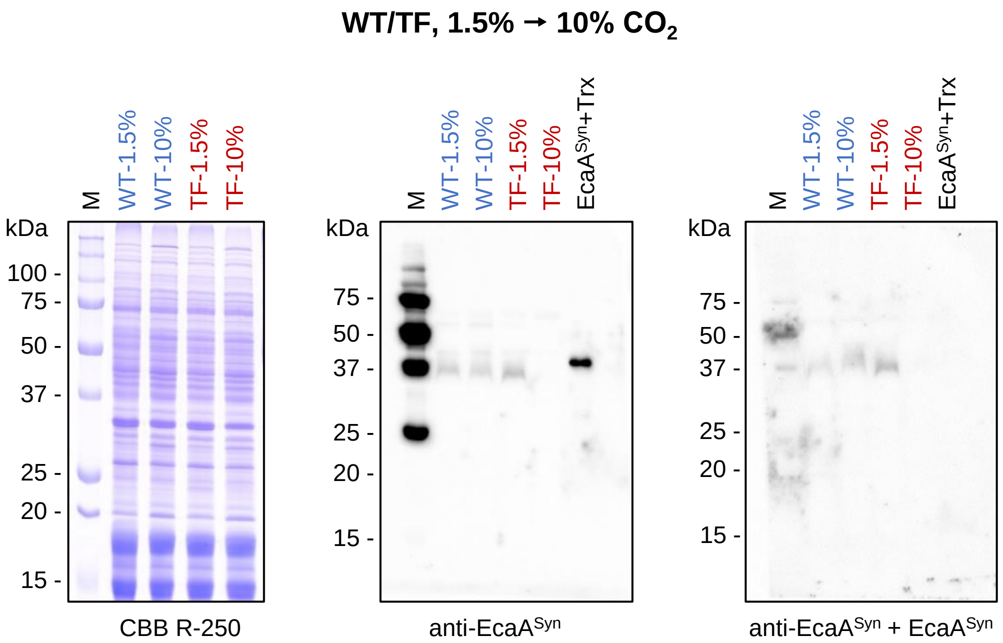
WT/TF, 1.5% → 10% CO_2
M
WT-1.5%
WT-10%
TF-1.5%
TF-10%
EcaA^{Syn}+Trx
kDa
100 -
75 -
50 -
37 -
25 -
20 -
15 -
CBB R-250
anti-EcaA^{Syn}
anti-EcaA^{Syn} + EcaA^{Syn}

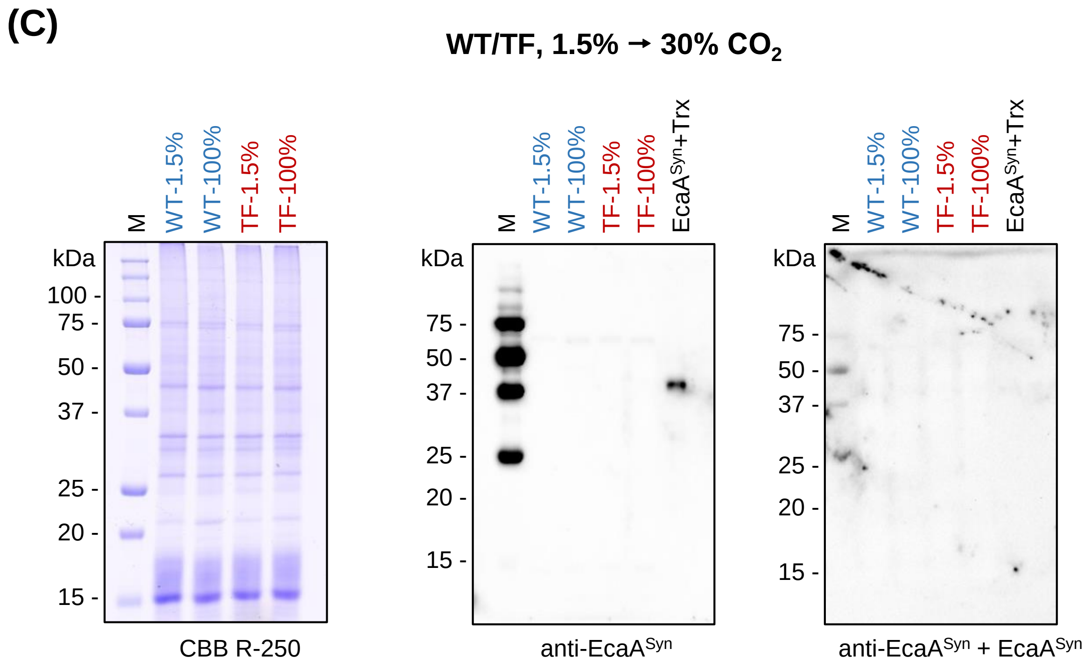

(C)
WT/TF, 1.5% → 30% CO_2
M
WT-1.5%
WT-100%
TF-1.5%
TF-100%
EcaA^Syn+Trx
kDa
100 -
75 -
50 -
37 -
25 -
20 -
15 -
CBB R-250
anti-EcaA^Syn
anti-EcaA^Syn + EcaA^Syn

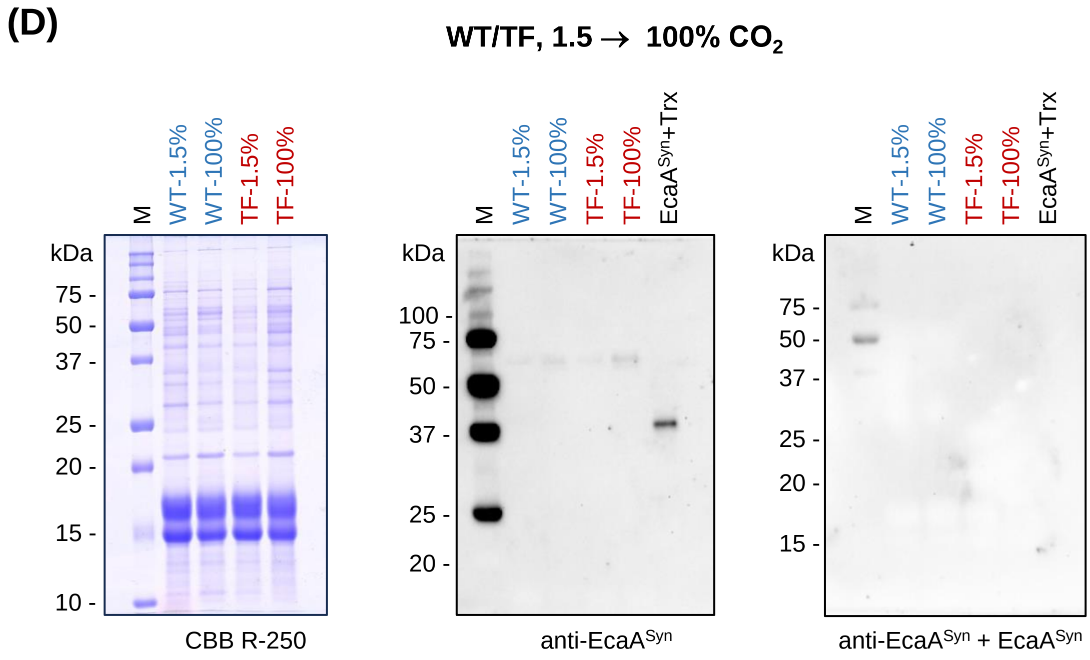

(D)
WT/TF, 1.5 → 100% CO_2
M
WT-1.5%
WT-100%
TF-1.5%
TF-100%
EcaA^Syn+Trx
kDa
100 -
75 -
50 -
37 -
25 -
20 -
15 -
10 -
CBB R-250
anti-EcaA^Syn
anti-EcaA^Syn + EcaA^Syn

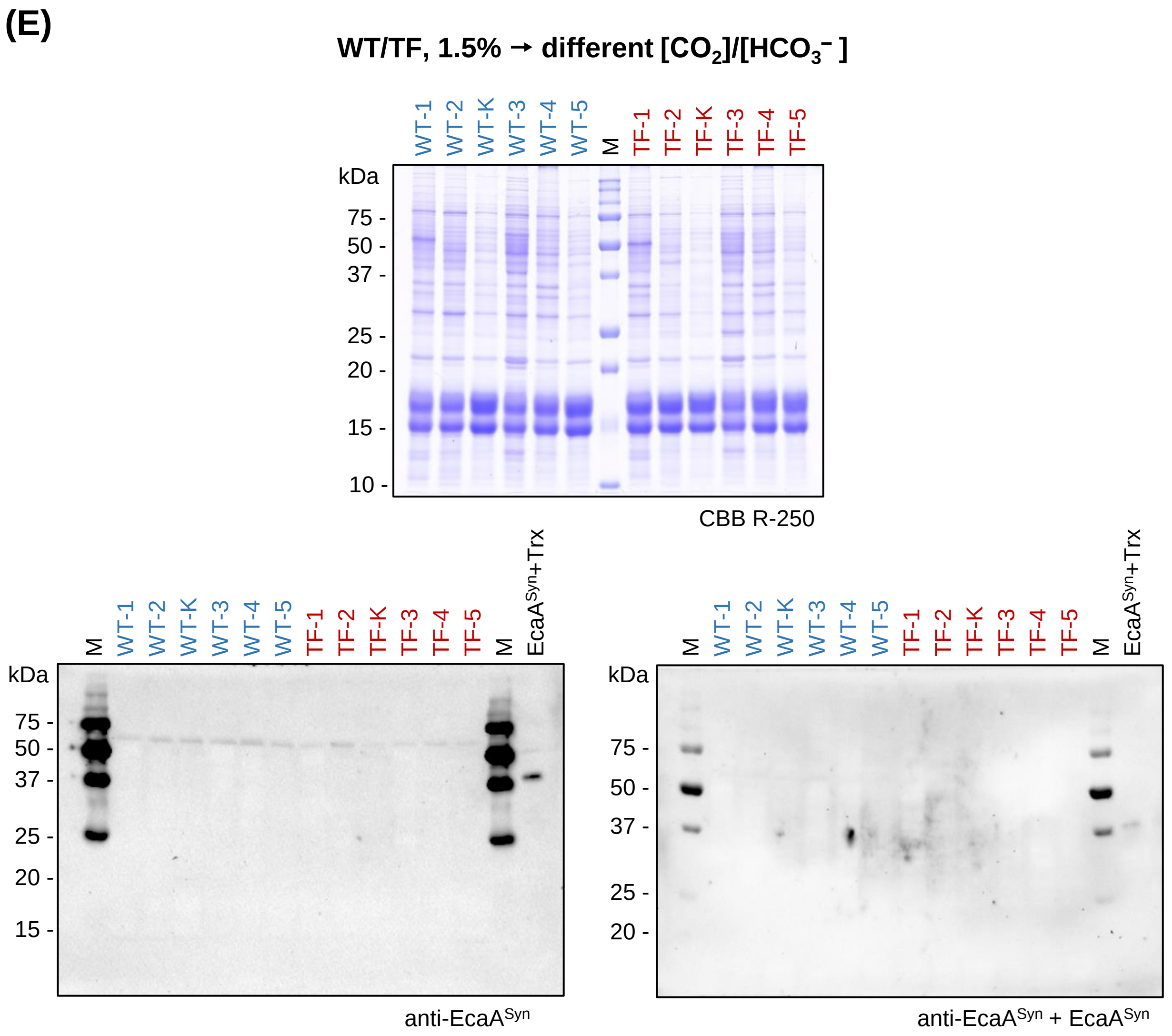

Figure S7. Immunolocalization of $EcaA^{Syn}$ in the soluble protein fraction of *Synechococcus* cells adapted to different CO_2 (A-D) or $[HCO_3^-]/[CO_2]$ (E) supply compared to the standard conditions (1.5% CO_2). Anti-$EcaA^{Syn}$ – the membrane treated with antibodies against recombinant $EcaA^{Syn}$ from *S. elongatus*. Anti-$EcaA^{Syn}$ + $EcaA^{Syn}$ – test for signal specificity; membrane was treated with antibodies preliminarily depleted in the presence of an excess of recombinant $EcaA^{Syn}$. The protein load per lane was 7.5 µg; load of recombinant $EcaA^{Syn}$+Trx – 2.5 ng. Signals were visualized using chemiluminescent solutions, and exposure times ranged from 1 to 4.5 min. Variants of experimental conditions corresponding to the samples at panel (E): K – BG-11, pH 7.5, 1.5% CO_2; 1 – BG-11, pH 7.5, without bubbling; 2 – BG-11, pH 7.5, 0.04% CO_2; 3 – BG-11 with 50 mM HCO_3^-, pH 9.5, without bubbling; 4 – BG-11 with 50 mM HCO_3^-, pH 9.5, 0.04% CO_2; 5 – BG-11 with 50 mM HCO_3^-, pH 9.5, 1.5% CO_2. Other designations: $EcaA^{Syn}$+Trx – affinity purified recombinant $EcaA^{Syn}$ protein fused with thioredoxin (41.6 kDa), positive control; WT/TF – samples corresponding, respectively, to wild-type *Synechococcus* cells and that of transformant with constitutive expression of the L^{Cya}-Eca^{Cya} protein; M – molecular weight marker.

(A)

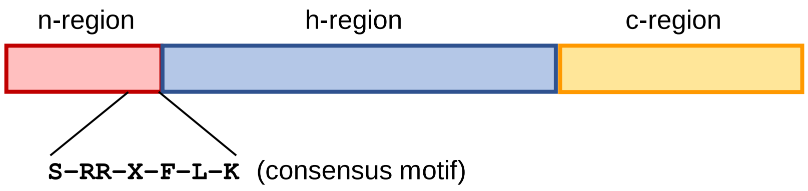

(B)

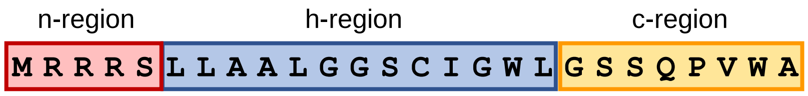

(C)

```
                                               SRRXFLK
E.coli TorA_CAD6017311.1     MNNNDLFQA-SRRRFLAQLGGLT----VAGMLGPSLLTP-----RRATAAQAATDAVISK   50
Syn IDIP-A_Q31L64.2          MSES-MF---SRRDFL--LGGTA----LAGTL---LLDSFGDWRRRAEAAEGEVNLYSGR   47
Syn Fe-SBP_WP_231621388.1    MSES-MF---SRRDFL--LGGTA----LAGTL---LLDSFGDWRRRAEAAEGEVNLYSGR   47
Syn GPM_ABB56976.1           MRF-------SRRRFLQSSLGAAATTGLAGTLAAGGQ----AQTRSTPVRKRSV-LVLGA   48
Syn PBP_ABB58296.1           MFL-------SRRRFLQASVGLPGLA-LAGCRSADLQLLLPGNTVPAPILRR----FQDR   48
Syn CmpA_P39660.2            MNE---FQPVNRRQFLFTLGATAA--------SAILLKGCGN--PPSSSGGGTSSTTQPT   47
Syn SHHK_ABB57846.1          MKL--------RRQFLLFGVGTT----FTAIAAISLSDVITN-TRLSESSREEVQSLLQR   47
Syn EcaA_AAC44830.1           MR---------RRSLLAALGGSC--IGWLGSSQPVWASA--DWDYSRRRGPRQWAKLDPA   47
```

Figure S8. The difference in a structure of the *n*-region of EcaA^{Syn} signal peptide between *S. elongatus* PCC 7942 and the classical signal sequences for transfer through the CM *via* the Tat translocation pathway. (A) General features of Tat signal peptides [Palmer & Stansfeld 2020]; (B) Structure of signal peptide of EcaA^{Syn} protein; (C) Alignment of *N*-terminal sequence of EcaA^{Syn} (EcaA_AAC44830.1) with that of *E. coli* TorA protein, as well as with a number of *Synechococcus* proteins with putative Tat-dependent signal peptide sequence. The position of the characteristic amino acid consensus motif is shown. The position of an essential phenylalanine residue is underlined. NCBI ac. nos. are added to the protein's names. The signal peptide sequences of the proteins in (A) and (B) are shown according to Phobius online tool [Käll et al. 2004] at http://phobius.sbc.su.se. The amino acid alignment (C) was performed using the Clustal V algorithm of MegAlign module of Lasergene v. 12.3.1 software package (DNAStar Inc., Madison, WI, USA).

References to Figure S8:

Palmer, T.; Stansfeld, P. J. Targeting of proteins to the twin-arginine translocation pathway. *Mol. Microbiol.* **2020**, *113*, 861–871. doi: 10.1111/mmi.14461

Käll, L.; Krogh, A.; Sonnhammer, E.L.L. A combined transmembrane topology and signal peptide prediction method. *J. Mol. Biol.* **2004**, *338*, 1027–1036. doi: 10.1016/j.jmb.2004.03.016
