# Supplementary material for: The Freshwater Cyanobacterium Synechococcus elongatus PCC 7942 Does Not Require an Active External Carbonic Anhydrase
Source: Plants (Basel). 2024 Aug 20;13(16):2323. doi: 10.3390/plants13162323 (PMC11360081; doi:10.3390/plants13162323)
Supplement: Supplementary file 1 [file plants-13-02323-s001.zip › Supplementary Tables_Kupriyanova_R1.pdf]

Table S1. Changes in mRNA levels of genes associated with C_i uptake systems in experimental variants **3 hour** after transferring cells from standard (BG-11, 1.5% CO_2) to experimental growth conditions.

**Cultivation mode**	***cmpA***		***sbtA***		***ndhF3***		***ndhF4***	
	**WT**	**TF**	**WT**	**TF**	**WT**	**TF**	**WT**	**TF**
№1, BG-11, no bubbling	24000 ± 890	31000 ± 1140	3900 ± 125	2400 ± 97	270 ± 14	320 ± 9	0.8 ± 0.05	1.1 ± 0.04
№2, BG-11, bubbling with 0.04% CO_2	106000 ± 114000	7800 ± 400	8200 ± 420	5000 ± 120	360 ± 13	280 ± 9	1.3 ± 0.1	1.1 ± 0.03
№3, 50 mM $NaHCO_3$, no bubbling	11 ± 2	2.0 ± 0.1	110 ± 3	70 ± 1.6	8 ± 0.2	10 ± 0.6	0.4 ± 0.1	0.4 ± 0.05
№4, 50 mM $NaHCO_3$, bubbling with 0.04% CO_2	2.7 ± 0.9	0.5 ± 0.1	2.8 ± 0.5	2.1 ± 0.2	2.3 ± 0.1	2.5 ± 0.1	0.7 ± 0.05	0.8 ± 0.01
№5, 50 mM $NaHCO_3$, bubbling with 1.5% CO_2	2.5 ± 0.1	2.1 ± 0.3	1.8 ± 0.5	1.8 ± 0.1	1.6 ± 0.2	2.0 ± 0.1	1 ± 0.1	0.8 ± 0.02

At least, a threefold change in the amount of the corresponding mRNA was considered a reliable change in gene expression (denoted by color).

Table S2. Changes in mRNA levels of genes associated with C_i uptake systems in experimental variants **6 hour** after transferring cells from standard (BG-11, 1.5% CO_2) to experimental growth conditions.

**Cultivation mode**	***cmpA***		***sbtA***		***ndhF3***		***ndhF4***	
	**WT**	**TF**	**WT**	**TF**	**WT**	**TF**	**WT**	**TF**
№1, BG-11, no bubbling	10000 ± 900	9500 ± 800	3000 ± 105	1600 ± 45	110 ± 2	103 ± 4	0.7 ± 0.06	0.8 ± 0.06
№2, BG-11, bubbling with 0.04% CO_2	55000 ± 2400	230 ± 25	7300 ± 450	3000 ± 102	260 ± 7	110 ± 9	1.1 ± 0.06	1.0 ± 0.04
№3, 50 mM $NaHCO_3$, no bubbling	23 ± 2	4.1 ± 0.4	530 ± 39	390 ± 8.9	20 ± 1	17 ± 0.7	0.5 ± 0.04	0.4 ± 0.02
№4, 50 mM $NaHCO_3$, bubbling with 0.04% CO_2	7.4 ± 1.9	2.0 ± 0.3	8.1 ± 1.6	5.5 ± 0.1	7.6 ± 0.3	8.8 ± 0.5	1.0 ± 0.06	1.2 ± 0.03
№5, 50 mM $NaHCO_3$, bubbling with 1.5% CO_2	6.6 ± 2.4	3.3 ± 0.2	1.9 ± 0.5	2.3 ± 0.2	1.2 ± 0.1	2.0 ± 0.2	0.7 ± 0.07	1.0 ± 0.06

At least, a threefold change in the amount of the corresponding mRNA was considered a reliable change in gene expression (denoted by color).

Table S3. Assembling of genetic constructs based on pTrc99a vector.

**Primers pairs**	**Sequence (5'→ 3')**	**PCR fragment***	**Genetic construct**	**Target protein**
ecaA^{Syn}-F-*Nco*I	ACCATGGGCCGGCGATCG	*Nco*I_*L*^{Syn}-*ecaA*^{Syn}_*Bam*HI	pTrc99::*L*^{Syn}-*ecaA*^{Syn}	L^{Syn}-EcaA^{Syn}
ecaA^{Syn}-R-*Bam*HI	GGATCCTTAGGCTTGCAGTGG			
L^{torA}-F-*Nco*I	CCATGGACAATAACGATCTCTTTCAG	*Nco*I_*L*^{torA}_*Eco*RI	pTrc99::*L*^{torA}-*ecaA*^{Syn}	L^{TorA}-EcaA^{Syn}
L^{torA}-R-*Eco*RI	GAATTCCGCAGTCGCACG			
ecaA^{Syn}-F-*Eco*RI	GAATTCAGTGCAGACTGGGAC	*Eco*RI_*ecaA*^{Syn}_*Bam*HI		
ecaA^{Syn}-R-*Bam*HI	GGATCCTTAGGCTTGCAGTGG			
ecaA^{Cya}-F-*Nco*I	CCATGGAAAGCACTAAAGTTATCC	*Nco*I_*L*^{Cya}-*ecaA*^{Cya}_*Bam*HI	pTrc99::*L*^{Cya}-*ecaA*^{Cya}	L^{Cya}-EcaA^{Cya}
ecaA^{Cya}-R-*Bam*HI	GGATCCGTTATTTATTGTCTTTGACAG			
L^{torA}-F-*Nco*I	CCATGGACAATAACGATCTCTTTCAG	*Nco*I_*L*^{torA}_*Eco*RI	pTrc99::*L*^{torA}-*ecaA*^{Cya}	L^{TorA}-EcaA^{Cya}
L^{torA}-R-*Eco*RI	GAATTCCGCAGTCGCACG			
ecaA^{Cya}-F-*Eco*RI	GAATTCGAAAAAACAACTCATAGTTGG	*Eco*RI_*ecaA*^{Cya}_*Bam*HI		
ecaA^{Cya}-R-*Bam*HI	GGATCCGTTATTTATTGTCTTTGACAG			

Generated sites for restriction endonucleases on the 5'-ends of primers are underlined.

* *L*^{Syn}-*ecaA*^{Syn} – nucleotide sequence for full-length EcaA protein of *S. elongatus* PCC 7942 with the native leader peptide; *ecaA*^{Syn} – nucleotide sequence for mature form of EcaA of *Synecococcus*, without leader peptide; *L*^{Cya}-*ecaA*^{Cya} – nucleotide sequence for full-length EcaA protein of *Cyanothece* sp. ATCC 51142 with the native leader peptide; *ecaA*^{Cya} – nucleotide sequence for the mature form of EcaA of *Cyanothece*, without leader peptide; *L*^{torA} – nucleotide sequence for leader peptide of TorA protein of *E. coli*.

Table S4. Nucleotide sequences of the synthetic oligonucleotides used as primers for RT-qPCR and semi-quantitative RT-PCR.

Organism	CyanoBase gene ID	Primer name	Sequence (5'→ 3')	Reference
Cyanothece sp. ATCC 51142	*cce_4328*	qCya-*ecaA*-F<br>qCya-*ecaA*-R	TGTAGCGAGAATGTGATCTGG<br>GGTCTAGCATTCATCGGGTATAG	Kupriyanova et al. 2019
S. elongatus PCC 7942	Synpcc7942_1388	qSyn-*ecaA*-F<br>qSyn-*ecaA*-R	GACAGAACACCACTGGATTACC<br>AGCTTCCCAGAAACCATTCC	Kupriyanova et al. 2018
	Synpcc7942_1488	qSyn-*cmpA*-F<br>qSyn-*cmpA*-R	TCGCAACTTCTTTAACGTG<br>TTGTAGGGATAGGAGACACTG	Woodger et al. 2003
	Synpcc7942_1475	qSyn-*sbtA*-F<br>qSyn-*sbtA*-R	AATATGCTCAGCAAGAGTCTG<br>CTTTACGCAGCTCACTAATTC	Woodger et al. 2003
	Synpcc7942_2091	qSyn-*ndhF3*-F<br>qSyn-*ndhF3*-R	CTACACGCCAATCCTTTATC<br>GCATCAGGAAGAAGACACTG	Woodger et al. 2003
	Synpcc7942_0309	qSyn-*ndhF4*-F<br>qSyn-*ndhF4*-R	CAATGGCAGCTGTTAGTCAC<br>CGATCGATGTAGAAGTCGTAG	Woodger et al. 2003
	Synpcc7942_0289	qSyn-*secA*-F<br>qSyn-*secA*-R	AGGTCAAGCAGTTCGTCTATC<br>TCTTTGAGGTCGTAGGCAATC	Kupriyanova et al. 2018
	Synpcc7942_2331	qSyn-*petB*-F<br>qSyn-*petB*-R	CTGATCCGCTCCATCCACC<br>GTTGCCTGACCGACGCTTT	Luo et al. 2019
	Synpcc7942_0626	qSyn-*ilvD*-F<br>qSyn-*ilvD*-R	CGGCGCTGCGGCTCAATAT<br>ATCATCGGCGGCGACAACC	Luo et al. 2019
	Synpcc7942_2252	qSyn-*ppc*-F<br>qSyn-*ppc*-R	GCCTCAAGCTCTCCTATATTC<br>GAATCAGCTTGAGATCTTCG	Woodger et al. 2003

Table S4. (continued)

Organism	CyanoBase gene ID	Primer name	Sequence (5’→ 3’)	Reference
S. elongatus PCC 7942	Synpcc7942_1473	qSyn-*ndhD5*-F	ACACTCGAGAATATCCTGAAACC	current study
		qSyn-*ndhD5*-R	TCGCTCTGGGCAACTTAATC	
	Synpcc7942_1264	qSyn-*nha2*-F	TTGGCGGGATTGACGATTAC	current study
		qSyn-*nha2*-R	ACTGATGTCGGTGTTGATGG	
	Synpcc7942_2359	qSyn-*nha3*-F	GGGCTTGATTGCTCAGTTTG	current study
		qSyn-*nha3*-R	TTCAGTTCTTCGCCCTTCTC	
	Synpcc7942_0991	qSyn-*pxcA*-F	TTGTCCTGCTGTTGGTCTTG	current study
		qSyn-*pxcA*-R	AAATTGCCTCTGGGTCTTGG	
	Synpcc7942_1656	qSyn-*katG*-F	GGGCCAGACTTCAACTATCAG	current study
		qSyn-*katG*-R	GTCTTGGCTATCGGTCATCAG	
	Synpcc7942_1214	qSyn-*GSHPx*-F	TCCACTCGTTTCGATGTCAG	current study
		qSyn-*GSHPx*-R	GCTGCGTAAAGAGGATGTTG	
	Synpcc7942_2309	qSyn-*2cys*-F	ATTCCAGACGATCAAGCTATCC	current study
		qSyn-*2cys*-R	GTAATTTCGGTCGGGCAAAC	
	Synpcc7942_2180	qSyn-*prxQA1*-F	TCGCAATTCCGAGGCAAGAC	current study
		qSyn-*prxQA1*-R	TAGGAATCACGGAAGCTGCAAG	
	Synpcc7942_0642	qSyn-*prxQB*-F	AGGTCAGCAAACGCTATG	current study
		qSyn-*prxQB*-R	ACTGCGGTGAAGTTAGC	

References to Table S4

Kupriyanova, E.V.; Sinetova, M.A.; Mironov, K.S.; Novikova, G.V.; Dykman, L.A.; Rodionova, M.V.; Gabrielyan, D.A.; Los D.A. Highly active extracellular α-class carbonic anhydrase of *Cyanothece* sp. ATCC 51142. *Biochimie* **2019**, *160*, 200–209. doi: 10.1016/j.biochi.2019.03.009

Kupriyanova, E.V.; Sinetova, M.A.; Bedbenov, V.S.; Pronina, N.A.; Los, D.A. Putative extracellular α-class carbonic anhydrase, EcaA, of *Synechococcus elongatus* PCC 7942 is an active enzyme: a sequel to an old story. *Microbiology* **2018**, *164*, 576–586. doi: 10.1099/mic.0.000634

Luo, X., Li, J.; Chang, T.; He, H.; Zhao, Y.; Yang, X.; Zhao, Y.; Xu, Y. Stable reference gene selection for RT-qPCR analysis in *Synechococcus elongatus* PCC 7942 under abiotic stresses. *Biomed. Res. Int.* **2019**, 7630601. doi: 10.1155/2019/7630601

Woodger, F.J.; Badger, M.R.; Price, G.D. Inorganic carbon limitation induces transcripts encoding components of the CO_2-concentrating mechanism in *Synechococcus* sp. PCC7942 through a redox-independent pathway. *Plant Physiol.* **2003**, *133*, 2069–2080. doi: 10.1104/pp.103.029728
